# Continuous MYD88 Activation Is Associated With Expansion and Then Transformation of IgM Differentiating Plasma Cells

**DOI:** 10.3389/fimmu.2021.641692

**Published:** 2021-05-04

**Authors:** Catherine Ouk, Lilian Roland, Nathalie Gachard, Stéphanie Poulain, Christelle Oblet, David Rizzo, Alexis Saintamand, Quentin Lemasson, Claire Carrion, Morgane Thomas, Karl Balabanian, Marion Espéli, Marie Parrens, Isabelle Soubeyran, Mélanie Boulin, Nathalie Faumont, Jean Feuillard, Christelle Vincent-Fabert

**Affiliations:** ^1^ UMR CNRS 7276/INSERM U1262 CRIBL, University of Limoges, and Hematology Laboratory of Dupuytren Hospital University Center (CHU) of Limoges, Limoges, France; ^2^ UMR CANTHER « CANcer Heterogeneity, Plasticity and Resistance to THERapies » INSERM 1277-CNRS 9020 UMRS 12, University of Lille, Hematology Laboratory, Biology and Pathology Center, CHU de Lille, Lille, France; ^3^ Institut de Recherche Saint-Louis, EMiLy, INSERM U1160, University of Paris, Paris, France; ^4^ Pathology Department, Hospital University Center of Bordeaux, Bordeaux, France; ^5^ Laboratory of Pathology, Institut Bergonié, Bordeaux, France

**Keywords:** MYD88 L265P mutation, lymphoplasmacytic lymphoma/Waldenstrom’s macroglobulinemia, IgM secretion, monoclonal Ig peak, B-cell lymphoma, plasma cell

## Abstract

Activating mutations of *MYD88* (*MYD88^L265P^* being the far most frequent) are found in most cases of Waldenström macroglobulinemia (WM) as well as in various aggressive B-cell lymphoma entities with features of plasma cell (PC) differentiation, such as activated B-cell type diffuse large B-cell lymphoma (DLBCL). To understand how MYD88 activation exerts its transformation potential, we developed a new mouse model in which the MYD88^L252P^ protein, the murine ortholog of human MYD88^L265P^, is continuously expressed in CD19 positive B-cells together with the Yellow Fluorescent Protein (Myd88^L252P^ mice). In bone marrow, IgM B and plasma cells were expanded with a CD138 expression continuum from IgM^high^ CD138^low^ to IgM^low^ CD138^high^ cells and the progressive loss of the B220 marker. Serum protein electrophoresis (SPE) longitudinal analysis of 40 Myd88^L252P^ mice (16 to 56 weeks old) demonstrated that ageing was first associated with serum polyclonal hyper gammaglobulinemia (hyper Ig) and followed by a monoclonal immunoglobulin (Ig) peak related to a progressive increase in IgM serum levels. All Myd88^L252P^ mice exhibited spleen enlargement which was directly correlated with the SPE profile and was maximal for monoclonal Ig peaks. Myd88^L252P^ mice exhibited very early increased IgM PC differentiation. Most likely due to an early increase in the Ki67 proliferation index, IgM lymphoplasmacytic (LP) and plasma cells continuously expanded with age being first associated with hyper Ig and then with monoclonal Ig peak. This peak was consistently associated with a spleen LP-like B-cell lymphoma. Clonal expression of both membrane and secreted µ chain isoforms was demonstrated at the mRNA level by high throughput sequencing. The Myd88^L252P^ tumor transcriptomic signature identified both proliferation and canonical NF-κB p65/RelA activation. Comparison with *MYD88^L265P^* WM showed that Myd88^L252P^ tumors also shared the typical lymphoplasmacytic transcriptomic signature of WM bone marrow purified tumor B-cells. Altogether these results demonstrate for the first time that continuous MYD88 activation is specifically associated with clonal transformation of differentiating IgM B-cells. Since MYD88^L252P^ targets the IgM PC differentiation continuum, it provides an interesting preclinical model for development of new therapeutic approaches to both WM and aggressive MYD88 associated DLBCLs.

## Introduction

Waldenström’s macroglobulinemia (WM) is an incurable indolent B-cell lymphoma of the elderly accounting for less than 5% of B-cell lymphomas with, as unique characteristics, a serum IgM peak and primary medullary localization of lymphoplasmacytic cells that exhibit continuous differentiation from mature B lymphocytes to IgM secretory plasma cells (1). Secondary lymphoid organ infiltration and/or a leukemic phase is found in 20% cases. Other manifestations include neuropathy, cryoglobulinemia, skin rash, cold-agglutinin hemolytic anemia, and amyloidosis (2). The discovery of the activating mutation of *MYD88* (*MYD88^L265P^* being the far most frequent) in more than 90% of WM cases contributed to the concept that this entity is genetically distinct from other B-cell lymphomas (3, 4). Being present in 50% of IgM monoclonal gammopathies of undetermined significance (MGUS), *MYD88* mutations are most likely a primary event in WM (5). Considered as secondary genetic events, activating mutations of *CXCR4* (CXCR4^S338X^ or CXCR4^WHIM^), a receptor implicated in migration and bone marrow (BM) homing of leucocytes, are found in 30% of WM cases (6). Additional mutations of *CD79b*, *ARID1A* or *TP53* have been reported (7).

Despite these advances, WM pathophysiology is incompletely understood. Its treatment remains challenging and the exact role of *MYD88* mutations in the emergence of lymphoplasmacytic B-cell clones is not known (7, 8). Indeed, *MYD88* mutations are also found in 30% of activated B-cell type diffuse large B-cell lymphomas (ABC-DLBCL), more than half of primary cutaneous DLBCLs, leg type, and many DLBCLs at immune-privileged sites but not in plasma cell myelomas, even IgM types (9). It should be noted that IgM expression is a surrogate marker of ABC-DLBCLs (10). Moreover, all these aggressive B-cell tumors associated with MYD88, which often exhibit morphological features of plasma cell (PC) differentiation, are all associated with expression of the PC differentiation marker IRF4. MYD88 protein is the canonical adapter for inflammatory signaling pathways to downstream members of the Toll-like receptor (TLR) and interleukin-1 receptor (IL-1R) families. Forming the myddosome complex, MYD88 binds IL-1R or TLR family members to IRAK kinases family. IRAK activation leads to activation of the NF kappa B (NF-κB) transcription factor and interferon 3 and 7 regulatory factors (IRF3 and 7). MYD88^L265P^ constitutively increases formation of the myddosome complex with downstream NF-κB activation (3, 11, 12). Experimentally, MYD88^L265P^ is essential for survival of ABC-DLBCL and WM cell lines (3, 11). A recent publication suggests the involvement of HOIP and LUBAC-dependent NF-κB activation in the transformation potential of MYD88 activation in a mouse model (13). The current published mouse models with continuous MYD88 activation in the B-cell lineage develop aggressive clonal B-cell lymphomas that resemble human ABC-DLBCLs (13–15). Although discussed by Jo et al. in the HOIP/LUBAC activation context, no IgM peak was reported in these models. Therefore, the question of a direct role for MYD88 in the development of a lymphoplasmacytic lymphoma with monoclonal IgM secretion is still open. Recently, K Schmidt et al. reported a mouse model in which MYD88 activation was responsible for an indolent lymphoproliferative disorder resembling to IgM monoclonal gammopathy of unknown significance (IgM MGUS), the asymptomatic preclinical stage of WM (16).

Here, we present a new mouse model in which the MYD88^L252P^ protein, the murine ortholog of human MYD88^L265P^, is continuously expressed in the B-cell lineage together with Yellow Fluorescent Protein (YFP) (Myd88^L252P^ mice). We show that these mice first developed early expansion of CD93^neg^ IgM PCs with an increase in both IgM secretion and bone marrow relocalization of IgM B-cells. Moreover, these mice also had increased percentages of IgM^high^ CD138^low^ and IgM^low^ CD138^high^ cells with a CD138 expression continuum between both cell types. Then, these mice developed an oligoclonal or clonal IgM lymphoplasmacytic-like B-cell lymphoma together with a serum IgM monoclonal peak. These tumors had marked transcriptomic similarities to WM but they were located in the spleen and exhibited significant increased proliferation. Despite differences between Myd88^L252P^ LP-like B-cell tumors and WM, our results demonstrate that the MYD88 transformation potential is strongly associated with a shift in B-cell peripheral differentiation toward plasma cells with IgM secretion. These results help explain why MYD88 activation is found in most WM and in various aggressive B-cell lymphomas with IgM PC differentiation engagement such as ABC DLBCLs.

## Materials and Methods

### Generation of the Transgenic Mouse Line Myd88^L252P^

The transgenes (cDNA) *Myd88^WT^*-IRES-*Yfp* and *Myd88^L252P^*-IRES-*Yfp* were synthetized (Genecust, Dudelange, Luxembourg) and inserted into the pcDNA3.1 vector. Sequences of these transgenes are given in **Supplementary Materials and Methods**. The *Myd88^L252P^*-IRES-*Yfp* insert was cloned into the pROSA26-1 vector (17) containing a LoxP-flanked region, consisting of a stop cassette and the *Neomycin* gene (18). The transgene *Myd88^L252P^*-IRES-*Yfp* was inserted downstream from the stop cassette. JM8 embryonic stem (ES) cells were transfected with the targeting vector according to a previously described protocol (19). Targeted ES cells were screened for homologous recombination by PCR. Genomic DNA (gDNA) extraction was performed using an in house protocol and consisted of gDNA precipitation with absolute ethanol. Twenty nanograms gDNA were used for each PCR (primer sequences in **Supplementary Materials and Methods**) using LongAmp^®^ *Taq* DNA Polymerase (New England Biolabs, Ipswich, MA) according to the manufacturer’s recommendations. Recombined ES cells were injected into C57BL/6J blastocysts which were transferred into foster mothers to obtain chimeric mice (Myd88^L252P-flSTOP^ mice).

### Mice

Cd19^Cre^ mice (20) and mice carrying the *Myd88^L252P^*-IRES-*Yfp* allele were crossed to induce the expression of the transgene in B cells (Myd88^L252P^ mice). Offspring were routinely screened by PCR using specific primers for insertion of the transgene (**Supplementary Materials and Methods**). Animals were housed at 21–23°C with a 12-hour light/dark cycle. All procedures were conducted under an approved protocol according to European guidelines for animal experimentation (French national authorization number: 8708503 and French ethics committee registration number APAFIS#14581-2018041009469362 v3).

### Cell Transfection and NF-κB Dependent Dual-Luciferase Reporter Assay

A20 cells (5.10^6^) were co-transfected with 5 µg of either empty pcDNA3.1, pcDNA3.1-*Myd88^WT^* or pcDNA3.1-*Myd88^L252P^* vectors, plus 100 ng pRL-TK *Renilla luciferase* expression vector (Promega Corporation, Madison, WI) and 5 µg of either the 3X-κB-L vector with three copies of the major histocompatibility complex (MHC) class I κB element or its mutated counterpart, the 3X-mutκB-L vector (21) using Amaxa L013 program (AMAXA Biosystems, Cologne, Germany). Twenty four hours after transfection, cells were lysed and luciferase activities were measured using the Dual-Luciferase Reporter Assay System and the Turner Designs TD-20/20 Luminometer (Promega Corporation, Madison, WI).

### Sera Analyses

Serum was obtained from blood collected retro-orbitally. Specific ELISA and serum electrophoresis assay were performed as previously described (19, 22).

### Flow Cytometry and *In Vivo* Proliferation Assays

In order to collect BM cells of Cd19^Cre^ and Myd88^L252P^ mice, femurs from both hind legs were rinsed with PBS and sternum was gently crushed and cells filtered on a nylon meshwork that was rinsed with PBS. Spleen cells from Cd19^Cre^ and Myd88^L252P^ were filtered through a sterile nylon membrane. Blood samples were collected retro-orbitally. Red cells were lysed by RBC Lysis Buffer (Biolegend, San Diego, CA). Cell suspensions were resuspended at 4°C in a labeling buffer (PBS, 1% BSA, 2mM EDTA) and labeled with fluorescent conjugated monoclonal antibodies listed in **Supplementary Materials and Methods**. Labeled cells were analyzed using a BD Fortessa SORP flow cytometer (BD Bioscience France, Le Pont de Claix, France). Results were analyzed using Kaluza Flow Cytometry software 1.2 (Beckman Coulter, Brea, CA).

### Immunohistochemistry

Paraffin embedded tissue sections (5µm) were deparaffinized as follows: slides were immersed successively in xylene twice for 3 min, 3 times for 3 min in 100% ethanol, once for 3 min in 95% ethanol and 3 times in PBS for 5 min. Then, slides were immersed in citrate buffer pH7 and heated 4 times for 5 min 40 sec in a microwave at 800W. Image acquisition was performed with the Nanozoomer 2.0RS Hamamatsu and NDP.scan software (812 Joko-cho, Higashi-ku, Hamamatsu City, 431-3196, Japan). Quantification of Ki67 nuclear labelling was performed with the imageJS and the Ki67 module: http://imagejs.org/?http://module.imagejs.googlecode.com/git/mathbiol.chromomarkers.js&http://module.imagejs.googlecode.com/git/ki67 (23).

### Gene Expression Profiling

A series of seven mice (three Cd19^Cre^ and four Myd88^L252P^) was studied in parallel with bone marrow purified tumor B-cells from a series of 11 patients with *MYD88^L265P^* WM (series 1) as well as lymph nodes from a series of 58 patients: 19 *MYD88^WT^* chronic lymphocytic leukemia, 15 *MYD88^L265P^* WM, 12 *MYD88^wt^* Nodal marginal zone lymphoma, 5 *MYD88^wt^* WM, 4 follicular lymphoma and 3 patients with benign follicular hyperplasia (series 2, **Supplementary Tables 1** and **2**). Approval of this protocol was obtained from the local IRB of the CHRU of Lille (CSTMT043)*. MYD88* and *CXCR4* mutational status was determined as previously described (6). Total mRNA was extracted from whole infiltrated tissues and purified B-cells as reported (24). For humans and mice, RNA amplification and hybridization onto microarrays were performed on an Affymetrix Human Genome U133 Plus 2.0 Array and on an Affymetrix Gene Atlas system^®^ with the MoGene-2_1-st-v1 Affymetrix chip (Affymetrix, Santa Clara, CA) respectively according to a previously described protocol (25) (GEO accession number GSE138273). Bioinformatic analyses are detailed in **Supplementary Materials and Methods**.

### Repertoire Analysis

RNA was extracted from total spleen, and 1µg was used for sequencing. Transcripts were amplified by 5’RACE PCR using reverse primers hybridizing within either the membrane or secreted exon of the µ or γ genes. ProtoScript^®^ II Reverse Transcriptase (New England Biolabs, Ipswich, MA) was used for reverse transcription and amplicons were obtained using Phusion^®^ High Fidelity DNA Polymerase (New England Biolabs, Ipswich, MA) according to the manufacturer’s instructions. Primers used are listed in **Supplementary Materials and Methods**. Illumina sequencing adapter sequences were added by primer extension, and resulting amplicons were sequenced on an Illumina MiSeq sequencing system using MiSeq kit Reagent V2 500 cycles. Paired reads were merged using FLASH (26). Repertoire analysis was done using the IMGT/HighV-QUEST online tool (http://www.imgt.org/IMGT_vquest/vquest). The resulting output was parsed by in-house R script.

## Results

### Generation of a Mouse Model With Insertion of the Mouse Mutation Myd88 L252P Into the Rosa26 Locus

To study the effect of continuous MYD88 activation on B-cell differentiation, we created a transgene containing the mutant murine cDNA sequence of *Myd88* (*Myd88^L252P^*) which is orthologous to the human mutant sequence *MYD88^L265P^*, in frame with the Yellow Fluorescent Protein (*Yfp*) sequence and separated by an Internal Ribosome Entry Site (IRES) sequence (*Myd88^L252P^*-IRES-*Yfp*) (**Supplementary Figure 1A**). To validate this transgene, we checked that it induced expression of both MYD88^L252P^ and YFP proteins in the murine A20 B-cell line (**Supplementary Figures 1B, C**) and that it was responsible for constitutive NF-κB activation (**Supplementary Figure 1D**). The *Myd88^L252P^*-IRES-*Yfp* insert was cloned into the pROSA26.1 vector (17) (**Supplementary Figure 1E**). In this construct, the insert was subcloned downstream from a *Neomycin*-STOP cassette flanked by LoxP sites. Chimeric mice were intercrossed to obtain stable germinal transmission of the *Myd88^L252P^*-IRES-*Yfp* transgene (Myd88^L252P-flSTOP^ mice). Myd88^L252P^ *^-flSTOP^* and Cd19^Cre^ mice were crossed. Mice with both transgenes (Myd88^L252P^ mice) were then studied, with their age matched Cd19^Cre^ littermates as controls (Cd19^Cre^ LMC). Specific B-cell expression of the transgene was found in more than 90% of blood and spleen B cells compared to virtually no expression in the T-cell compartment (**Supplementary Figure 1F**). As expected and as evidence of NF-κB activation, Myd88^L252P^ splenocytes over-expressed the NF-κB target gene *Tnfaip3* at the mRNA level (**Supplementary Figure 1G**).

### Serum Protein Electrophoresis Profiles Segregate Myd88^L252P^ Mice According to Age

**Figure 1:**
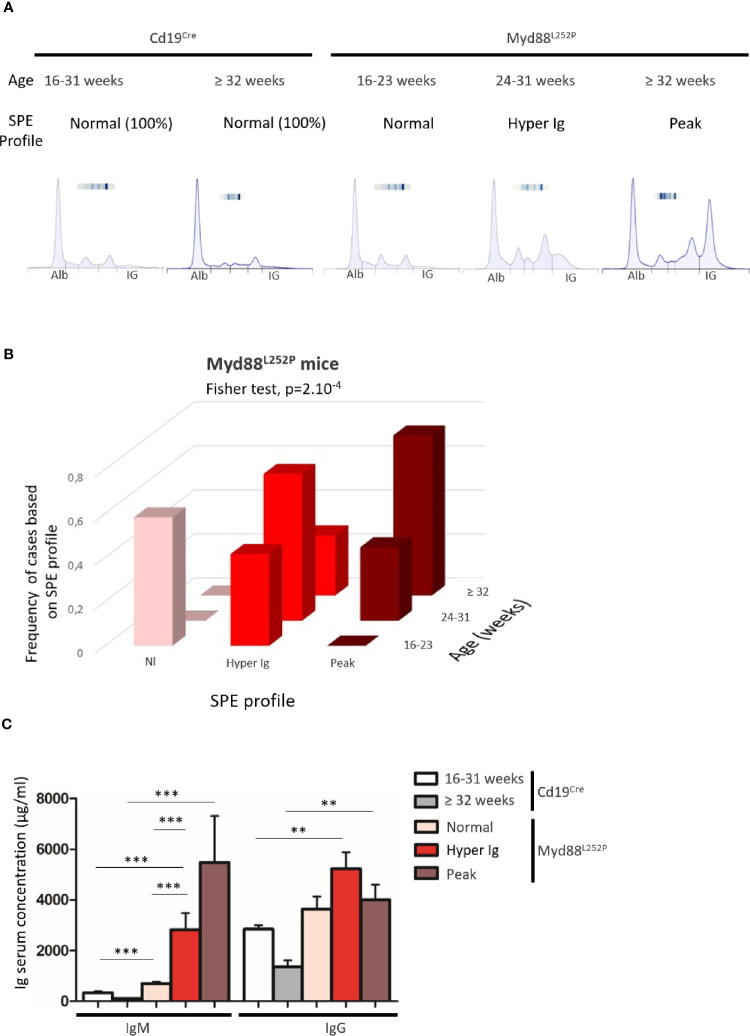
Myd88^L252P^ transgenic mice exhibited serum IgM hypergammaglobulinemia and then monoclonal IgM peaks when ageing. **(A)** Examples of serum protein electrophoresis of Cd19^Cre^ mice (respectively 16 and 36 weeks-old) and three Myd88^L252P^ mice (16, 24 and 36 week-old) with normal, polyclonal hypergammaglobulinemia (hyper Ig) and monoclonal Ig peaks respectively. **(B)** Frequencies of cases according to SPE profile and age for Myd88^L252P^ mice (n = 40). **(C)** IgM and IgG serum levels in Cd19^Cre^ and Myd88^L252P^ mice. For Cd19^Cre^ (n = 15), Myd88^L252P^ (n = 36). Results are expressed as the mean ± SEM. Mann Whitney test p-value < 0.01 and < 0.001 are symbolized by ** and *** respectively.

As a first exploratory step, serum protein electrophoresis (SPE) was systematically performed on a series of 40 Myd88^L252P^ mice and 26 age matched Cd19^Cre^ LMCs. As shown in **Figure 1A** three SPE profiles were seen: normal, polyclonal hyper gammaglobulinemia (hyper Ig) and a monoclonal Ig peak. All Cd19^Cre^ LMCs exhibited normal SPE regardless of their age. In other words, SPEs with hyper Ig or Ig peaks were found only in Myd88^L252P^ mice (**Figures 1A, B**). **Figure 1B** shows the relationship between the age of Myd88^L252P^ mice and the SPE profile. Young Myd88^L252P^ mice (16-23 weeks) had a normal or hyper Ig SPE profile. In contrast, most mice older than 32 weeks had an Ig peak. In between these two groups, 24 to 31 week old mice (middle age) had a hyper Ig or an Ig peak in 65% and 35% cases respectively (Fisher test, p=2.10^-4^). ELISA quantification of serum Ig revealed that young Myd88^L252P^ mice with a normal SPE exhibited a moderate IgM and IgG hyper Ig when compared to their Cd19^Cre^ LMC (**Figure 1C** and **Supplementary Figure 2**). Serum IgG levels of middle aged and old Myd88^L252P^ mice were variable when compared to their young counterparts. This was in contrast to serum IgM levels that were significantly increased in middle aged mice and even more so in old mice and correlated with the SPE profile and age (**Figure 1C** and **Supplementary Figure 2**).

These results first indicate that continuous MYD88 activation in B cells was associated with a global increase in Ig secretion. Second, age related occurrence of polyclonal hyper Ig and then monoclonal Ig peaks correlated with the increase in serum IgM levels. This suggests that, after a polyclonal expansion phase, aging of Myd88^L252P^ mice was associated with clonal restriction of IgM-secreting B-cells, very likely reflecting the MYD88^L252P^ B-cell transformation power in these cells. Therefore, these first results point to a strong relationship between MYD88^L252P^ and IgM-secreting B-cells.

### Myd88^L252P^ Bone Marrow IgM Plasma Cell Content Was Increased and Displayed a CD138 Expression Continuum

**Figure 2:**
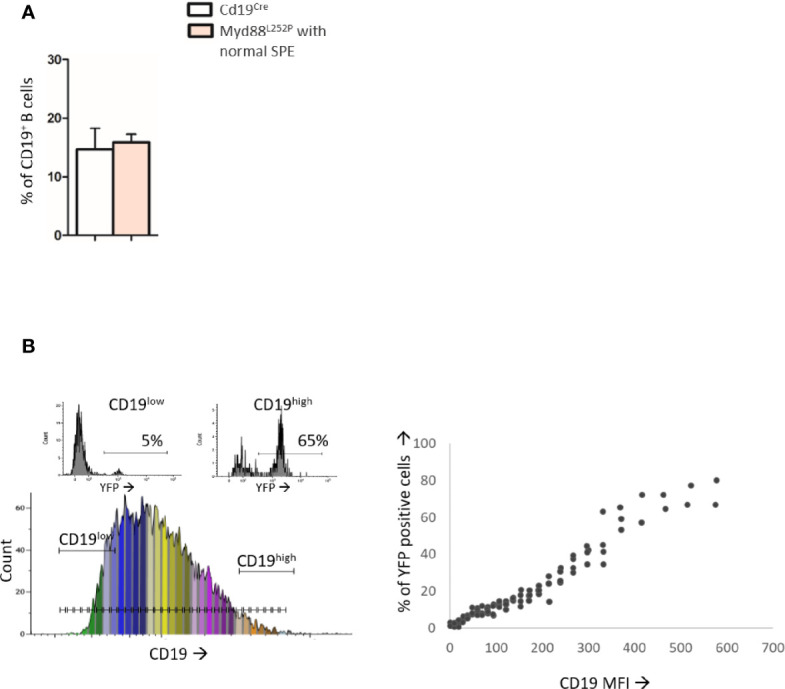
Analysis of B-cell differentiation in bone marrow from 16 week-old Cd19^Cre^ and Myd88^L252P^ mice. **(A)**: Percentage of CD19^pos^ and/or B220^pos^ B-cells in bone marrow from 16 week-old Cd19^Cre^ (n =9) and Myd88^L252P^ mice (n = 9). Results are expressed as the mean ± SEM. **(B)**: Flow cytometry analysis of the transgene expression according to CD19 expression levels (n=9). Left panel: CD19 monoparametric histogram sliced according to CD19 MFI intervals. For each CD19 MFI interval, the percentage of bone marrow B220^pos^ YFP positive cells was noted. Two examples of YFP monoparametric histograms are presented in the upper part, one for CD19^low^ B220^pos^ B-cells and one for CD19^high^ B220^pos^ B-cells with their respective percentages of YFP^pos^ cells Right panel: percentage of YFP^pos^ B220^pos^ B-cells (Y axis) according to CD19 MFI (X axis).

**Figure 3:**
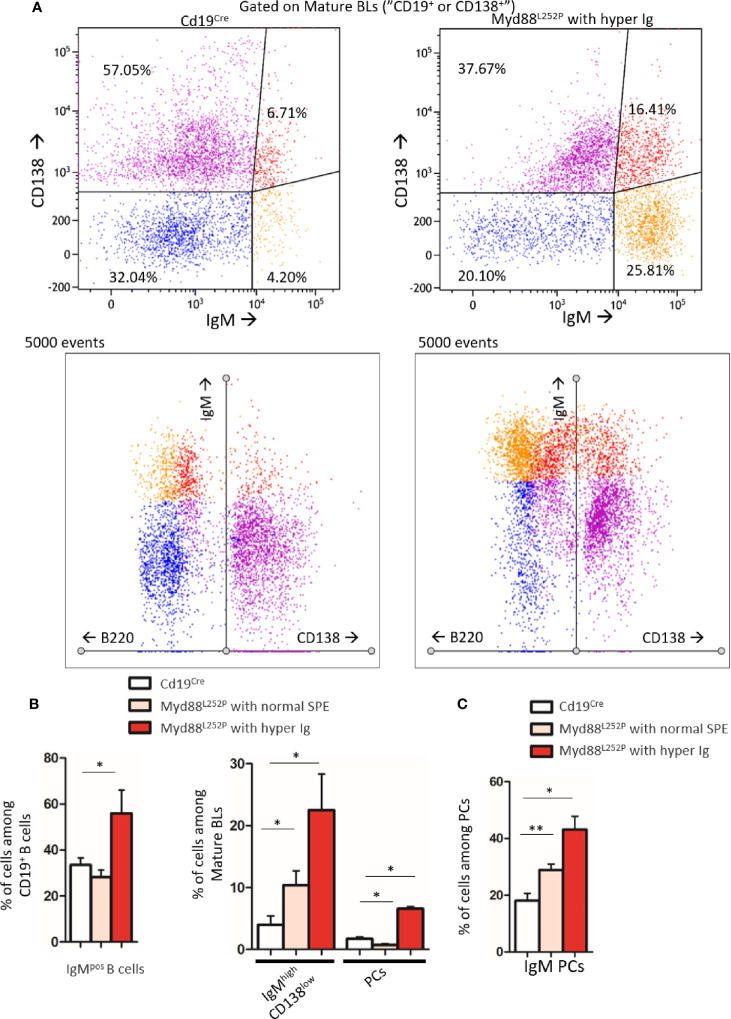
Increase in the IgM PC compartment in bone marrow from Myd88^L252P^ transgenic mice. **(A)** Example of bi and triple parametric flow cytometry histograms gated on mature B or plasma cells for expression of IgM, B220 and CD138 for Cd19^Cre^ and Myd88^L252P^ mice (left and right panels respectively). Upper panels: IgM^low or neg^ CD138^neg^, IgM^high^ CD138^low^, IgM^high^ CD138^high^ and IgM^low^ CD138^high^ cells are colored in blue, orange, red and purple respectively using a hinged quadstat of the Kaluza software and an IgM/CD138 2-dimensional plot. The hinged quadstat was set-up for one Cd19^Cre^ mouse. Lower panels: triple parametric histograms using the radar function of the Kaluza software. Note the CD138 expression continuum on Myd88^L252P^ bone marrow B-cells that correlated with a progressive decrease in B220 expression. This CD138 expression continuum was virtually absent in Cd19^Cre^ mice. **(B)** Percentages of total IgM^pos^ B cells and IgM^high^ CD138^low^ pre PCs and B220^low^ CD138^high^ PCs in bone marrow from 16-24 week-old Cd19^Cre^ (n =6) and Myd88^L252P^ mice with normal SPE or hyper Ig (n = 6 and n = 3 respectively). Results are shown as the mean ± SEM. Mann Whitney test p-value < 0.05 is symbolized by *. **(C)** Percentages of IgM^pos^ CD138^high^ PCs among total PCs in bone marrow from 16-31 week-old Cd19^Cre^ (n =6) and Myd88^L252P^ mice with normal SPE or hyper Ig (n = 6 and n = 3 respectively). Results are shown as the mean ± SEM. Mann Whitney test p-value < 0.05 and < 0.01 are symbolized by * and ** respectively.

As shown in **Figure 2A**, Myd88^L252P^ bone marrow global B-cell content was comparable to that of Cd19^Cre^ LMCs in terms of percentages in 16 week old mice with normal SPE. Transgene expression was mainly found in Myd88^L252P^ CD19^high^ B-cells. Indeed, with an on/off effect, percentages of YFP positive cells (*i.e* of LoxP rearranged cells) was directly correlated with CD19 mean fluorescence intensity (**Figure 2B**). Comparison of CD19^cre^ LMC and Myd88^L252P^ mice did not reveal any significant bone marrow B-cell increase with age. However older Myd88^L252P^ mice had increased levels of IgM^pos^ CD19^high^ B-cells (**Figures 3A, B**, left panel and **Supplementary Figure 3** for the gating strategy). Strikingly, a CD138 expression continuum was clearly evident in a triple parametric B220/CD138/IgM histogram gated on mature B-cells and/or PCs in Myd88^L252P^ mice only (**Figure 3A**, lower panel see in red the decrease in B220 and the increase in CD138 expression). This CD138 expression continuum, that we recently showed to be characteristics of MYD88^L265P^ WM bone marrow tumor B-cells (Gayet et al, Cytometry B 2021), started from IgM^high^ CD138^low^ and ended at IgM^low^ CD138^high^ cells (**Figure 3A**, lower panel). This CD138 expression continuum was absent in Cd19^Cre^ LMCs. Consequently, Myd88^L252P^ mice showed increased percentages of both IgM^high^ CD138^low^ B-cells (most likely precursors of IgM PCs) and total bone marrow PCs (**Figure 3B**, right panel). Moreover, the proportion of bone marrow IgM PCs was significantly increased in young Myd88^L252P^ mice and even more in older Myd88^L252P^ mice (**Figure 3C**). Indeed, Myd88^L252P^ CD19^pos^/YFP^pos^ B cells tended to accumulate in the IgM^high^ B-cell compartment when compared to its Myd88^L252P^ CD19^pos^/YFP^neg^ counterpart (**Supplementary Figure 4**).

Altogether, these results indicate that transgene expression started in a minority of CD19^weak^ B-cell precursors and was mainly expressed at the latest stages of B-cell lymphopoiesis when CD19 expression was high. Evidence for bone marrow increase in both IgM^high^ CD138^low^ and PCs with the characteristic CD138 expression continuum may either suggest that the bone marrow of Myd88^L252P^ mice had the ability to home IgM PC precursors and PCs and/or a shift in peripheral B-cell differentiation toward IgM PC in Myd88^L252P^ mice.

### MYD88^L252P^ First Induced Peripheral Early Lymphoplasmacytic and Plasma Cells Expansion and Then B-Cell Transformation Into a Lymphoplasmacytic-Like Lymphoma

**Figure 4:**
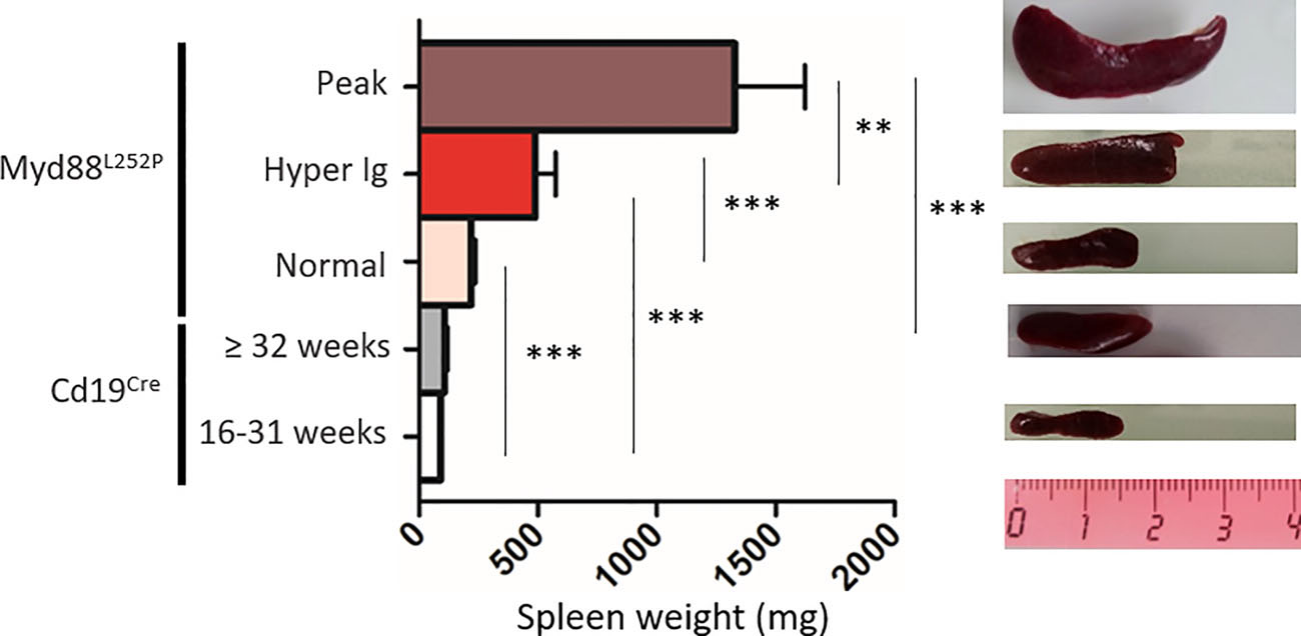
Myd88^L252P^ mice exhibited a progressively increasing splenomegaly consistently related to the SPE profile. Spleen size of Cd19^Cre^ and Myd88^L252P^ age-paired mice. Myd88^L252P^ mice were sacrificed together with at least one Cd19^Cre^ mouse of the same age. Left panel: distribution of spleen weights; right panel: examples of spleen macroscopy (Cd19^Cre^ n = 9 for 16-31 weeks-old and n = 16 for ≥ 32 weeks; Myd88^L252P^ with normal SPE: n = 9; with hyper Ig: n = 13; with Ig peak: n = 19). Results are given as the mean ± SEM. Mann Whitney test p-value < 0.01 and p-value < 0.001 are symbolized by ** and *** respectively.

Extended white blood cell differential was not altered and mice did not exhibit any palpable/visible peripheral lymphadenopathy (data not shown) regardless of the SPE profile. Young Myd88^L252P^ mice with normal SPE tended to have spleen enlargement when compared to their age related Cd19^Cre^ LMCs (**Figure 4**). Spleen enlargement was dramatically increased in Myd88^L252P^ mice with hyper Ig and even more so in those with an IgM peak, a feature that was most likely to related to B-cell transformation. Indeed, the B/T cell ratio was markedly increased in these latter mice (**Supplementary Figure 5**).

**Figure 5:**
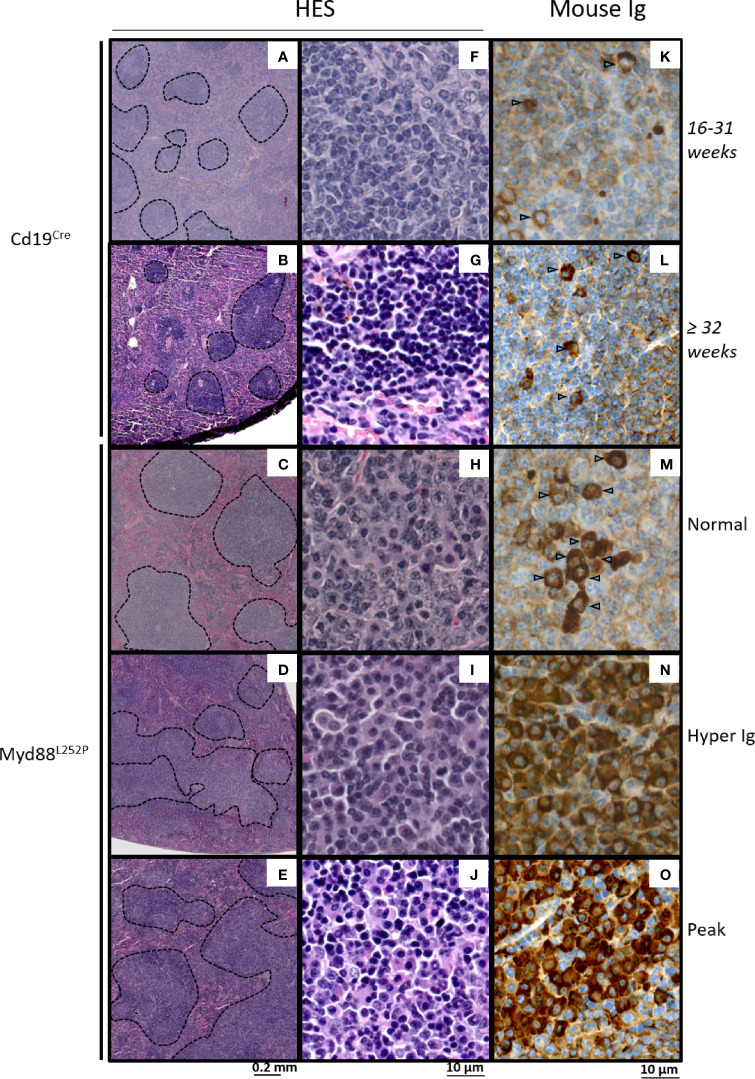
Morphological and immunophenotypic plasma cell engagement of Myd88^L252P^ tumors. Hematein eosin morphological aspect **(A–J)** and intracytoplasmic Ig labeling **(K–O)** of Cd19^Cre^ and Myd88^L252P^ spleens: one 16-31 week-old Cd19^Cre^ **(A, F, K)**, one ≥ 32 week-old Cd19^Cre^ **(B, G, L)** and three Myd88^L252P^ **(C, H, M)** for Normal group, **(D, I, N)** for Hyper Ig group and **E, J, O** for Peak group) mice are shown. Panels **(A–E)** at low magnification, Myd88^L252P^ tumors often exhibited a nodular pattern **(C–E)**. Panels **(F–J)**: at high magnification, most Myd88^L252P^ tumors had small B-cell aspects with marked lymphoplasmacytic engagement (panels **H–J)**. Panels **(K–O)** Myd88^L252P^ tumors with a lymphoplasmacytic aspect exhibited marked plasma cell differentiation as revealed by the presence of intracytoplasmic Ig in numerous cells (arrows) with various labeling intensities **(M–O)**.

While spleen histology of young Myd88^L252P^ mice was comparable to that of their Cd19^Cre^ LMCs, with an apparent normal spleen architecture, Myd88^L252P^ mice with hyper Ig or an Ig peak exhibited enlarged and congruent lymphoid nodules (**Figures 5A–E**). At high magnification, a marked lymphoplasmacytic aspect consisting of a mixture of small to large lymphocytes with numerous lymphoplasmacytic cells (LP cells) and PCs was noted in all Myd88^L252P^ mice whatever their SPE profile (**Figures 5F–J**). This spleen aspect was particularly striking for mice with Ig peaks, and was characterized by massive and diffuse infiltration of lymphoplasmacytic cells that evoked human B-cell lymphomas with features of PC differentiation, further called “LP-like lymphoma” or “LP-like tumors”. (see also the cytological imprint in **Supplementary Figure 6**). Presence of LP cells and PCs in Myd88^L252P^ spleen was invariably confirmed by immunohistochemistry after intracellular Ig labeling regardless of SPE status, with numerous LP cells and terminally differentiated PCs (cells with intermediate or strong intracytoplasmic Ig labeling respectively). Noteworthy, cell densities were markedly increased in Myd88^L252P^ mice with hyper Ig or with an Ig peak (**Figures 5K–O**).

**Figure 6:**
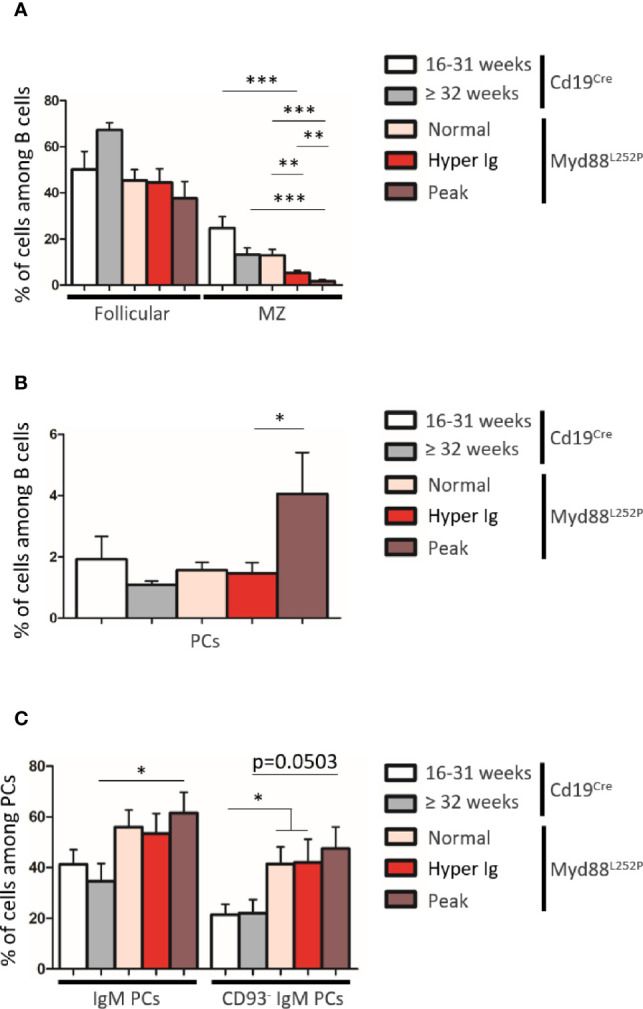
B-cell differentiation in spleens from Cd19^Cre^ and Myd88^L252P^ mice. **(A)** Percentage of Follicular B cells (CD21^pos^ CD23^high^) and marginal zone (MZ) B cells (CD21^high^ CD23^pos^). **(B)** Percentage of plasma cells (PCs, B220^low^ CD138^high^). **(C)** Percentage of IgM^+^ PCs and CD93^neg^ IgM PCs. Myd88^L252P^ mice (with normal SPE: n = 7; with hyper Ig: n = 10; with Ig peak: n = 9) were compared to Cd19^Cre^ mice LMCs (16-31 week and ≥ 32 week-old mice; n = 9 for each group) Results are presented as the mean ± SEM. Mann Whitney test p-value < 0.05, p-value < 0.01 and p-value < 0.001 are symbolized by *, ** and ***.

Based on B-cell expression of CD21 and CD23, frequencies of CD21^pos^ CD23^high^ follicular B-cells were not significantly altered in Myd88^L252P^ mice regardless of their SPE status (**Figure 6A** and **Supplementary Figure 7**). In contrast, a decrease of CD21^high^ CD23^pos^ marginal zone B-cells was observed. This cell content nearly disappeared in Myd88^L252P^ mice with an Ig peak. Only total PCs were increased in these mice (**Figure 6B**). However, among total spleen PCs, percentages of IgM PCs were increased in a similar manner in all Myd88^L252P^ mice no matter what their SPE status was (**Figure 6C** and **Supplementary Figure 7**). Moreover IgM PCs were predominantly CD93^neg^ suggesting that they belonged to the proliferating PC compartment (27) (**Figure 6C** and **Supplementary Figure 7**).

Therefore, morphological and immunophenotypic results indicated that continuous MYD88 activation was associated with continuous peripheral IgM PC differentiation very early on and that these LP and PC subsets continuously expanded with age first being associated with hyper Ig and then with an Ig monoclonal peak and a LP-like lymphoma aspect in the spleen.

### Proliferation Index of Myd88^L252P^ LP-Like Tumors Was Moderately Increased in Myd88^L252P^ Tumors With an LP-Aspect

**Figure 7:**
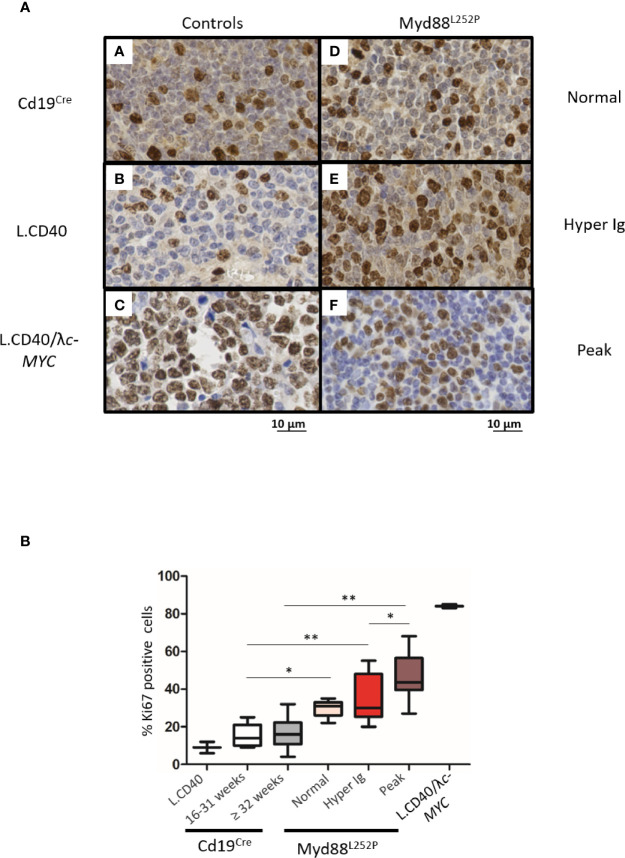
Intermediate increase in Myd88^L252P^ tumor proliferation rate. **(A)** Examples of Ki67 labeling in spleen sections from three controls (panels A-C) and three Myd88^L252P^ mice (panels D-F, n=28). Controls were Cd19^Cre^ LMCs (panel A, n = 5 for 16-31 week-old mice and n = 8 for ≥ 32 week-old mice), L.CD40 mice (panel B) with indolent splenic lymphomas of marginal zone B-cells (n=2), as well as L.CD40/λc-Myc mice (panel C, n=2) with a ABC-DLBCL lymphomas (24, 28). The three Myd88^L252P^ tumors (panels D-F) are one representative example of each group defined according the SPE profile (normal n = 5, hyper Ig n = 8 and Ig peak n = 15). Here, L.CD40 mice were used as a model of indolent B-cell lymphoma with a low proliferation index while L.CD40/λc-Myc mice were a model of aggressive B-cell lymphoma with a high proliferation index. **(B)** Quantification of Ki67 labeling. Box plots represent the median and quartile of percentages of Ki67 positive cells. Mann Whitney test p-value < 0.05, p-value < 0.01 are symbolized by * and ** respectively.

To better study these Myd88^L252P^ LP-like tumors, we compared their Ki67 proliferation index to that their of Cd19^Cre^ LMCs as well as to L.CD40, L.CD40/Λ*c-MYC* mice as controls. L.CD40 mice are a model of marginal zone spleen B cell indolent lymphomas without plasma cell differentiation but with NF-κB activation (28). L.CD40/Λ*c-MYC* mice are a model of ABC-DLBCLs with both c-Myc and NF-κB activation in B-cells (24). Very few Ki67 positive cells were seen outside germinal centers in spleen sections from Cd19^Cre^ mice (**Figure 7A** panel A). The Ki67 index was weak in L.CD40 mice (**Figure 7A** panel B). By contrast, the vast majority of cells from L.CD40/Λ*c-MYC* tumors were Ki67 positive (**Figure 7A** panel C). Being moderately increased in young mice with a normal SPE, the Ki67 proliferative index was further enhanced in mice with hyper Ig and even higher in those with an Ig peak, but never reached the proliferation index levels of L.CD40/Λ*c-MYC* tumors (**Figures 7A**, panels D–7 and **7B**). *In vivo* incorporation of BrdU was tested for four mice with an Ig peak and one with polyclonal hyper Ig. Results confirmed that the proliferation index was consistently increased (**Supplementary Figure 8**) albeit always less than 30% with this technique.

Altogether, these results show that MYD88^L252P^ expression in B-cells was responsible for progressive peripheral B-cell expansion related to an early increase in B-cell proliferation. Mice with an Ig peak clearly exhibited a lymphoproliferative disease with a marked increase in proliferation index but with features of an LP-like lymphoma such as the presence of numerous LP cells and PCs.

### Myd88^L252P^ Mice With Ig Peaks Developed IgM but Not Igg Monoclonal or Oligoclonal B-Cell Lymphomas With Expression of Both Membrane and Secretory Heavy Chain mRNA

**Figure 8:**
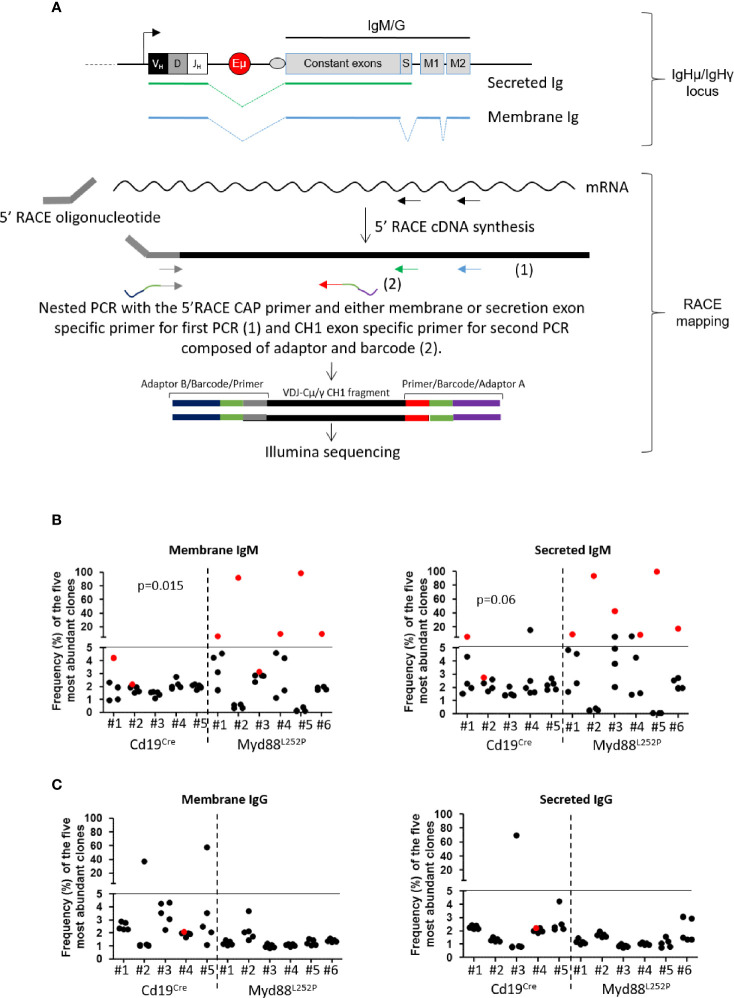
µ and γ heavy chain mRNA clonal abundance: **(A)** RACE PCR technique quantifying clonal mRNA abundance of membrane and secreted forms of mouse µ and γ heavy chains. *Ighμ/Ighγ* locus (upper panel): *Igh* locus with variable regions (VDJ), the enhancer Eµ and constant genes (for IgM or IgG). Box “S” represents the secreted exon used for the secreted form of Ig, “M1 and M2” represent the membrane exons for the membrane form of Ig. Green or blue dotted lines show RNA splicing respectively for secreted and membrane Ig. RACE mapping (lower panel): 5’RACE PCR followed by preparation of libraries for Illumina sequencing. First, we amplified cDNA between the primer specific for the membrane or the secreted form (black arrows) and the 5’RACE oligonucleotide. Amplicons for Illumina sequencing were obtained after two nested PCRs; the first with the 5’ Race CAP primer and either membrane (blue arrow) or secretion (green arrow) exon specific primer, and the second with the same 5’ primer and a CH1 exon specific primer (grey arrow). For sequencing, forward (grey) and reverse (red) primers used for the second PCR contained adaptors (blue and purple) and a barcode (orange); each barcode sequence was specific for one sample only. **(B, C)** Relative frequency of the five most abundant mRNA clones coding for membrane (left) and secreted (right) forms of µ **(B)** and γ **(C)** heavy chains for Cd19^Cre^ (n = 5) and Myd88^L252P^ (n = 6) mice. The most abundant clones are highlighted in red when VDJ sequences of the dominant membrane and secreted clones were identical. Myd88^L252P^ mice exhibited IgM but not IgG clonal expansion with expression of both secreted and membrane form of the µ chain. Wilcoxon’s test p-value are given in the figure.

Since Myd88^L252P^ mice had a global hyper Ig even if predominantly IgM, it was important to assess µ or γ chain clonality of tumor surface and secreted immunoglobulins at the molecular level. Six LP-like cases with monoclonal Ig peaks and five Cd19^Cre^ mice were studied. mRNA reverse transcription followed by RACE PCR with primers specific for either the membrane or secreted forms of mouse µ or γ heavy chains was performed, followed by high throughput sequencing (HTS) of the VDJ-Cµ or VDJ-Cγ region (**Figure 8A**). **Figures 8B, C** show the relative frequency of the five most abundant mRNA clones for the µ or γ heavy chains respectively. RACE PCR and HTS results indicate that Myd88^L252P^ mice developed clonal or oligoclonal B-cell expansion with expression of both secreted and membrane forms of the µ heavy chain that had the same VDJ-Cµ clonal rearrangement (**Figure 8B**), without any bias in terms of V segment usage (not shown). The same RACE PCR technique with primers specific for either the membrane or secreted form of the mouse γ heavy chain did not identify any significant B-cell clonal expansion in Myd88^L252P^ tumors (**Figure 8C**).

These results indicate that, despite initial IgM and IgG hyper Ig in young Myd88^L252P^ mice, MYD88^L252P^ specifically promoted IgM B-cell lymphomagenesis with clonal expression of both membrane and secreted µ chain isoforms identical VDJ gene rearrangements. These results are in full agreement with the lymphoplasmacytic aspect Myd88^L252P^ tumors and the persistent IgM plasma cell differentiation continuum.

### Transcriptomic Signature of Myd88^L252P^ LP-like Tumors Revealed NF-κB RelA Activation, Proliferation and Plasma Cell Differentiation and Overlapped With Waldenström’s Macroglobulinemia Gene Expression Profile (GEP)

To explore the transcriptomic signature of Myd88^L252P^ LP-like tumors in conditions similar to those of most studies on human B-cell lymphomas and to look for common features with WM, we selected a short series of massively invaded Myd88^L252P^ spleen tumors with monoclonal Ig peaks. Comparisons were first done with their Cd19^Cre^ LMCs, and then with WM patients.

**Figure 9:**
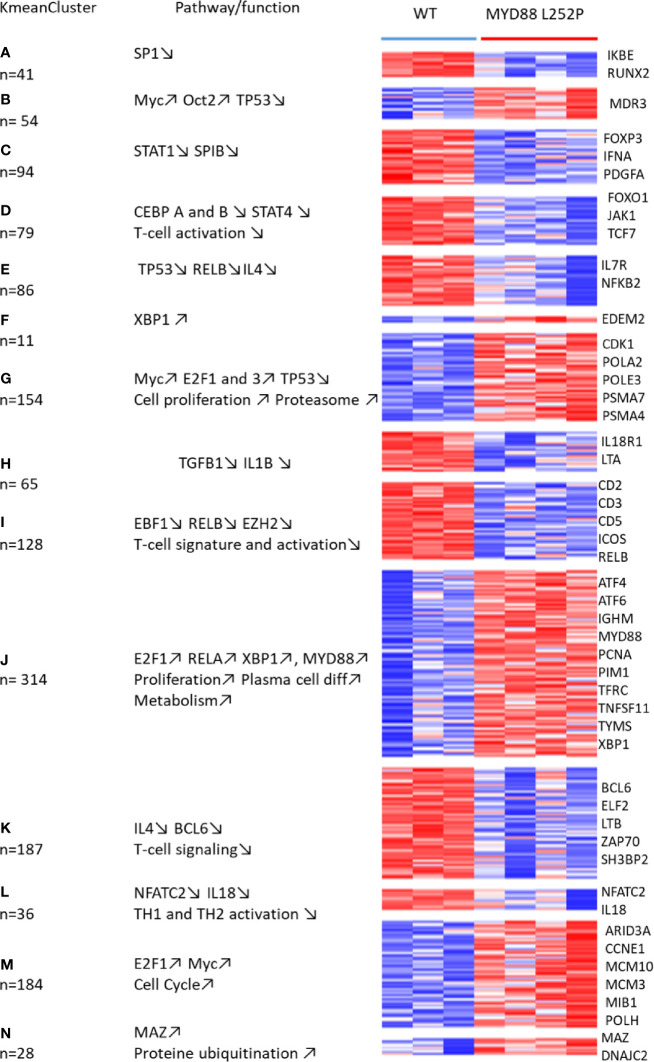
Whole transcriptome analysis of Cd19^Cre^ and Myd88^L252P^ mice: total mRNA was extracted from whole spleen tissues. Gene expression profiles were obtained using the MoGene-2_1-st-v1 Affymetrix chip. mRNA transcripts (3236) were selected to be differentially expressed using the Limma R package. Expressed genes that were too heterogeneous were eliminated, resulting in a final selection of 1515 genes. These genes were segregated into 40 Kmean clusters. The closest Kmean clusters were merged 2 by 2 according to their proximity by principal component analysis of the mean vectors. This was repeated until maximization of the absolute value of Chi2 (29). This resulted in 14 aggregated clusters. Functional annotation of the aggregated Kmean clusters was performed using the Ingenuity Pathway Analysis (IPA) Software. Annotated heatmap of the 1515 genes are segregated into the 14 aggregated clusters. Left: the aggregated Kmean clusters with the corresponding number of genes; middle: main pathways and or function identified with the IPA software; right: some relevant genes.

With a fold change of 2, a set of 1515 differentially expressed genes were found in Myd88^L252P^ spleen tumors when compared to spleens from Cd19^Cr^ *^e^* LMCs (**Supplementary Table 3**). To analyze this set of genes, we combined both K-mean and hierarchical clustering and principal component analysis, as already published (29). Following this methodology, deregulated genes in Myd88^L252P^ spleen tumors could be segmented into 14 classes of co-regulated genes (**Figure 9** with methodological details in **Supplementary Materials** and **Methods**, **Supplementary Figure 9** and **Supplementary Table 4**). Consistent with increased B/T ratios in Myd88^L252P^ LP-like tumors, expression of genes belonging to the T-cell lineage, as well as T-cell signaling and activation signatures was down-regulated in Myd88^L252P^ spleen tumors (**Figure 9**, see clusters I, K and L). Of note, RelB signature was associated with that of T-cells and was decreased in Myd88^L252P^ spleen tumors. In contrast, expression of genes belonging to the proliferation, *RelA* NF-κB activation pathway and plasma cell differentiation signatures were up-regulated (**Figure 9**, cluster J mainly as well as cluster B, G and M for proliferation).

**Figure 10:**
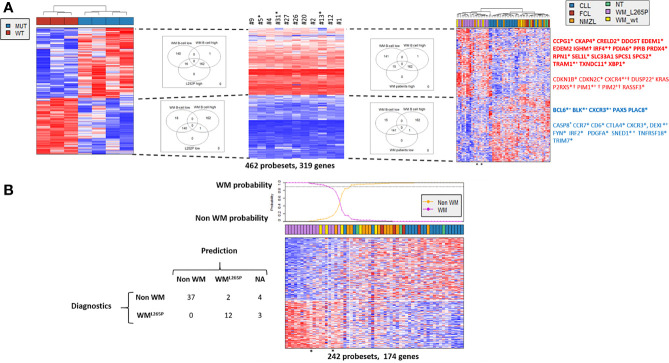
Comparison of gene expression profile (GEP) of Myd88^L252P^ mice and patients with WM or other indolent B-cell NHL. Affymetrix differential gene expression profiles (GEP) between four Myd88^L252P^ mice (MUT, n=4) and three Cd19^Cre^ mice (WT, n=3) were compared to the Affymetrix GEP of purified bone marrow tumor B-cells from 11 WM patients with the *MYD88^L265P^* mutation, resulting in selection of 462 probesets (319 genes). This selection was used on the Affymetrix transcriptome of an independent series of lymph node biopsies from 58 patients: 19 *MYD88^wt^* chronic lymphocytic leukemias (CLL), 15 *MYD88^L265P^* WM (WM_L265P), *12 MYD88^wt^* nodal marginal zone lymphomas (NMZL), 5 *MYD88^wt^* WM with IgM peaks (WM_WT), 4 follicular lymphomas (FCL) and 3 patients with follicular hyperplasia (NT). **(A)** Hierarchical clustering and heatmap of the 462 selected probesets for mice (left), purified bone marrow *MYD88^L265P^* WM B-cells (middle) and lymph nodes (right). Down and up-regulated genes are in blue and red respectively. Branches of down and up regulated genes in Myd88^L252P^ mice, *MYD88^L265P^* WM bone marrow B-cells and *MYD88^L265P^* WM lymph nodes are delineated by dashed lines. Venn diagrams of the intersections between the branches are shown, highlighting the consistency between branches across the different clustering. Some genes of interest are noted on the right. In bold are those of the plasma cell signature; *: genes in the predictor (see **Figure 7B**); +: genes reported by Hunter et al. in WM (30); † : genes of the ABC/GC DLBCL signature (31). **(B)**: Informativeness of *MYD88^L265P^* WM diagnosis using the 462 selected probesets defined in **Figure 8A**. The 462 probesets defined from MYD88^L252P^ mice and bone marrow tumor B-cells from *MYD88^L265P^* WM patients were used to predict *MYD88^L265P^* WM diagnosis (WM^L265P^ versus non WM) from other lymphomas within the series of 58 lymph node biopsies. Probabilities that each sample belongs to WM^L265P^ versus non WM group are indicated. The WM^L265P^ versus non WM or not attributed (NA) assignment is shown on the left.

To identify MYD88^L252P^ deregulated genes in common with those of WM patients having the *MYD88^L265P^* mutation, transcriptomes of Myd88^L252P^ tumors were compared to those of purified WM bone marrow B-cells from a series of 11 *MYD88^L265P^* WM patients. A subset of 319 coherently dysregulated genes in both Myd88^L252P^ LP-like tumors and WM tumor B-cells (163 up and 156 down, **Supplementary Tables 5, 6**) was extracted from the 1515 differentially expressed genes in Myd88^L252P^ LP-like tumors. Unsupervised hierarchical clustering showed a 95% coherency between the branches of up and down regulated genes in both WM tumor B-cells and LP-like Myd88^L252P^ spleen tumors (**Figure 10A**, left and middle panels and **Supplementary Tables 5, 6**). To check whether this Myd88^L252P^/WM signature could discriminate WM from other indolent NHLs, an independent series of 58 patients, including 15 *MYD88^L265P^* WM, five *MYD88^wt^* WM, 12 *MYD88^wt^* NMZLs and 19 *MYD88^wt^* CLL was analyzed (**Supplementary Tables 2, 7**). All *MYD88^L265P^* WM patients clustered together after unsupervised hierarchical clustering (**Figure 10A**, right panel and **Supplementary Tables 7, 8**). Also belonging to the *MYD88^L265P^* WM cluster were 3/5 (60%) *MYD88^wt^* WM and 2/12 (17%) *MYD88^wt^* NMZLs. We used the linear predicting score described by Wright et al. (31) to estimate the informativeness of the Myd88^L252P^/WM signature for WM diagnosis. As shown in **Figure 10B**, as set of 174 genes (84 up and 90 down, **Figure 10B**) was found to predict WM with over 90% probability (**Supplementary Tables 9, 10**).

The plasma cell signature was the main component of the Myd88^L252P^/WM GEP. Genes such as *EDEM1* and *2*, *IRF4* or *XBP1* were over-expressed while others such as *PAX5* or *BCL6* were markedly down-regulated (**Figure 10**). Consistently, functions revealed by Gene Set Enrichment analysis mainly corresponded to endoplasmic reticulum and Golgi apparatus (not shown). In accordance with results published by Hunter et al. (30), expression of genes such as *CXCR4*, *DUSP22*, *PIM1* and *2* or *TRAM1* was increased while expression of *SNED1* was decreased. Few genes of the Myd88^L252P^/WM signature overlapped with those of ABC/GC DLBCLs published by Wright et al. (31). These overlapping genes, corresponding only to those that are overexpressed in ABC DLBCLs, were *IRF4, IGHM, CXCR4, P2RX5, PIM1* and *PIM2.* In other words, the Myd88^L252P^/WM signature did not significantly overlap with that of GCB DLBCLs. Among other deregulated genes were cyclin kinase inhibitors *CDKN1B* (*p27^kip1^*) and *CDKN2C* (*p18/INK4AC*), mutations of the former being found in hairy cell leukemias (32). *RASSF3* and *KRAS* were also up-regulated. *RASSF3* belongs to the Ras association domain family (RASSF).

Altogether, the Myd88^L252P^ tumor signature highlights proliferation as well as canonical NF-κB p65/RelA activation (but not RelB), which is in agreement with the known fact that MYD88 activates the classical NF-κB pathway. The Myd88^L252P^ tumor signature also strikingly confirms that lymphoplasmacytic differentiation is at the heart of MYD88 related B-cell transformation in mice, a feature shared with WM tumors with the *MYD88^L265P^* mutation.

## Discussion

Different mouse preclinical models with continuous MYD88 activation in B-cells have been published; All but one demonstrate the B-cell transformation potential of MYD88 but without presenting evidence for a correlation between IgM B-cell LP and PC differentiation (13–15).. The first report was published by Knittel et al. (14). The authors generated a mouse model that allows B-cell conditional expression of the *Myd88^L252P^* allele from the endogenous *Myd88* locus. In this model, expression of *Myd88^L252P^* would be regulated in a manner similar to that of the wild type allele. At least three regulatory levels of MYD88 activity have been reported. The MYD88 regulatory region harbors various transcription binding sites such as those for NF-κB, IRF1, SP1 or STAT factors and it was shown that this gene is regulated by IL-6 (33), which suggests a role for its expression in either plasma cell differentiation or inflammation. An alternative splice variant of *MYD88*, *MYD88s*, lacks exon 2 and is unable to activate NF-κB. This variant is also able to form a heterodimer with the full length MYD88 protein, resulting in decreased formation of the myddosome complex (34). MYD88s is increased during sepsis and is thought to ensure robust termination of MYD88 dependent inflammation (35, 36). As a third regulation mechanism, hypomethylation and upregulation of MYD88 are important for NF-κB activation (37). MYD88 promoter demethylation is important in glioblastoma and is associated with increased MYD88 protein expression in lung cancers (38, 39). Mice from Knittel’s model occasionally developed DLBCLs when they aged. Therefore, the Knittel mouse model raises the question of the relationship between B-cell lymphomagenesis and regulation of the endogenous *Myd88* locus throughout B-cell life. In addition, this model also showed hyper Ig, even if polyclonal, which may suggest that expansion of Ig secreting B-cells could precede the lymphoma development. In that view, K Schmidt et al. recently published a mouse model with a transgene closed to the one of the Knittel’s model. But, after exploring the effect of MYD88 activation in three different Cre context, Cd19-Cre, Cγ1-Cre and Cd19-Cre^ERT2^, as well as analyzing the effect of NP-immunization, the authors concluded that MYD88 continuous activation promotes survival of long term IgM expressing B-cells with clonal restriction a monoclonal serum IgM paraprotein resulting in an IgM MGUS-like disorder (16).

To our knowledge, two papers, from Wang et al. and Sewastianik et al. reported the effect of the human MYD88^L265P^ protein in murine B-cells (15, 40). In the model of Wang et al, primary murine B-cells were retrovirally infected *ex vivo* before reinjection. In this model, an initial boost of B-cell proliferation was seen followed by B-cell apoptosis in a Bim-dependent manner. Importantly, no increase in immunoglobulin secretion was reported. Beside the fact that retroviral infection of B-cells may have its own interfering effects, this model raises the question of whether the human MYD88^L265P^ protein may have exactly the same activation properties on a mouse B-cell background as in human B-lymphocytes. In the model of Sewastianik et al, the human loxP-flanked-stop-*MYD88^L265P^* transgene was inserted downstream from the mouse *Collagen type I alpha 1 chain* (*Col1A1*) gene and MYD88^L265P^ expression was induced by crossing with *Aid^Cre^* mice (15). In addition to focal skin rashes, some WM features were noted such as expansion of lymphoplasmacytic cells and increased IgM serum levels. However, only DLBCL clonal transformation was seen. Because AID is mainly expressed in germinal center B-cells and because the promoter of the *Col1A1* gene is highly active in fibroblasts and osteoblasts (41), this model raises the question whether temporality and/or expression pattern could be important in MYD88 driven B-cell lymphomagenesis.

The *Rosa26* locus has been solidly proven to be valuable for expression of numerous oncogenes in the B-cell lineage (see reference (42) for discussion as well as the literature of the K Rajewsky’s group). By inserting our *Myd88^L252P^*-IRES-*Yfp* transgene in this locus, we forced continuous expression of the mutated MYD88 protein in a heterozygous-like context while respecting the native MYD88 activation pathways of mouse B-cells. Moreover, we were able to monitor our transgene expression by flow cytometry due to YFP. Thereby, we created a conditional Myd88^L252P^ mouse model closed to the one published by Jo et al. (13). However, these authors mainly focused their work on the synergy between MYD88 and the catalytic subunit HOIP which increases LUBAC ligase activity that in turns promotes NF-κB canonical activation; only four so-called CD19-cre-MYD88LP have been studied at the tumor stage.

Here, a longitudinal analysis of a series of 40 Myd88^L252P^ mice compared to their age matched Cd19^Cre^ LMCs demonstrated that IgM plasma cell expansion is at the heart of MYD88 dependent B-cell transformation. Indeed, by examining clonal restriction of IgM secreting B-cells, we first showed that ageing of Myd88^L252P^ mice was associated with polyclonal hyper Ig followed by monoclonal Ig peak due to increased serum IgM. Second, we provide evidence indicating that bone marrow relocalization of IgM B-cells, IgM^high^ CD138^low^ cells and IgM PCs was increased in Myd88^L252P^ mice with a CD138 expression continuum, that is a characteristic of WM tumor B-cells. Third, analysis of spleen morphology and spleen B-cell subsets by flow cytometry indicated that continuous MYD88 activation was associated very early with peripheral LP cell and CD93^neg^ IgM PC expansion and that these cell subsets were markedly increased at the time of the Ig monoclonal peak. Fourth, appearance of a monoclonal Ig peak was constantly associated with a B-cell lymphoma with marked features of lymphoplasmacytic differentiation, so-called Myd88^L252P^ LP-like lymphoma. Fifth, at the molecular level, Myd88^L252P^ specifically promoted IgM B-cell lymphomagenesis with mRNA clonal expression of both membrane and secreted µ (but not γ) chain isoforms. Finally, the Myd88^L252P^ tumor gene expression profile not only highlights the canonical NF-κB p65/RelA activation pathway and proliferation, but also strikingly shares an *Xbp1* centered lymphoplasmacytic B-cell differentiation signature with MYD88^L265P^ WM. This signature differentiates MYD88^L265P^ WM from other indolent B-cell tumors including marginal zone lymphomas. Our results contradict the conclusions of Sewastianik et al. (15), and, being in line with those of K Schmidt et al. (16), firmly demonstrate the specific transforming effect of MYD88 activation in IgM PC differentiating B-cells.

To establish the gene expression signature from bulk Myd88^L252P^ spleen tumors rather than from purified B-cells is matter of discussion. The very obvious disadvantage of working on bulk tumors is certainly that all mRNA species from all cell types present in the tissue are mixed together. Even if massively invaded, both stromal and other residual immune cells (T-cells, macrophages, dendritic cells…) persist constantly in the tumor. In these conditions, specifically assigning a given mRNA expression pattern to tumor cells is always hazardous. However, because tumors were immediately snap-frozen, all mRNA species are supposed to be well preserved without any significant experimental bias. On the other hand, working on purified cells may also induce artefacts since the abundance of different mRNA species may vary during the time of purification which can also stress the cells. Above all, in the specific case of Myd88^L252P^ spleen tumors, which exhibit an LP aspect with continuous PC differentiation, the key question would have been to choose the right negative selection marker. Indeed, tumor cells are phenotypically heterogeneous with variable expression of B220, surface Ig or CD138 for example. Should we have selected B220^high^ versus B220^low^ B-cells or CD138^low^ versus CD138^high^ cells? Rather than make wrong or partial choices concerning which tumor cells to purify, we chose to work on bulk Myd88^L252P^ spleen tumors with well-preserved total mRNA and to compare this bulk signature to that of WM, including purified WM bone marrow tumor B-cells.

One characteristic feature of Myd88^L252P^ B-cells was the strong reduction of the marginal zone B-cell compartment. While also characterized by continuous activation of NF-κB, the L.CD40 mouse model published by Hömig-Hözel et al. (28), in which B-cells are submitted to continuous CD40 activation signaling, showed expansion of marginal zone B-lymphocytes. This points out the differences between TLRs and CD40 in terms of NF-κB activation. Indeed, CD40 is able to activate both the classical and alternative pathways, i.e. to induce the nuclear translocation of RelA and RelB NF-κB containing complexes while TLRs only activate the classical pathway. The effect of both pathways as well as the strength of NF-κB activation on B-cell fate has been extensively discussed by Pillai et al. (43). In this review, the authors also indicate that BTK activation blocks the Notch signaling pathway that is essential for marginal zone B-cell differentiation. It turns out that Hunter et al. have shown that Myd88 is able to activate BTK in a BCR independent manner (30), which consequently could repress B-cell maturation toward the marginal zone B-cell lineage.

Gene expression profiles of LP-like Myd88^L252P^ tumors distinctly suggest the involvement of RelA rather than RelB. RelA, but not RelB, is clearly associated with EBV-dependent B-cell immortalization and with EBV-associated DLBCL tumors, which exhibit a phenotype close to that of ABC-DLBCLs (25). RelA is also essential for development of GC-derived PCs (44) and immunohistochemistry detected nuclear RelA in WM B-cells (45). Indeed, LP-like Myd88^L252P^ B-cell tumors shared strong overlaps with human WM in terms of gene expression profile. Even if a few genes were in common with the ABC-DBCL signature such as *IGHM, CXCR4* or *PIM1* and *PIM2*, the Myd88^L252P/^WM signature points to dysregulation of plasma cell differentiation as the keystone of MYD88 transforming physiopathology. It also suggests that *KRAS* activation could be important. *RASSF3* and *KRAS* itself were up-regulated in both Myd88^L252P^ B-cell tumors and human WM. Consistently, and in agreement with results from the group of Treon (30), we also found *RASSF6* overexpression in WM patients (NG and JF unpublished results). CXCR4, whose expression is increased in WM, may activate the RAS pathway through RasGAP-associated proteins (46). The most frequent mutations involve *KRAS* and *NRAS* genes in multiple myeloma (47). Even if such mutations have not been reported in WM (6), our results highlight the putative role of the Ras activating pathway in WM, which may lead to the design of novel therapies.

Despite similarities between WM and Myd88^L252P^ LP-like tumors such as serum Ig monoclonal peak, increase in IgM prePCs, PC bone marrow relocalization and marked lymphoplasmacytic differentiation of tumor cells some major differences exist. The first difference, is the predominant site of tumor involvement. Even if splenomegaly is found in 20% of bona fide WM patients, it is largely admitted that bone marrow is the primary tumor site. In contrast, Myd88^L252P^ LP-like mouse tumors mainly developed in the spleen. Physiologically, IgM PCs tend to reside mainly in the spleen whereas switched IgG PCs migrate to the bone marrow (48). This raises the question of why WM IgM tumor B-cells migrate to bone marrow. In that view, it is largely suspected that *CXCR4* mutation could play a role in bone marrow homing. Further studies could also evaluate the transforming potential of Myd88 by adoptive transfer of LP-like tumor B-cells. Whether the transferred tumor will retain the lymphoplasmacytic aspect would also be interesting.

Another significant difference is the presence of large cells and increased proliferation in Myd88^L252P^ LP-like mouse tumors. The increased proliferation index was an early event since it was also found in young Myd88^L252P^ mice with normal SPE. As we previously discussed (24), only three mouse models for indolent lymphomas of the spleen have been published, one mimicking TRAF3 inactivation, the second with constitutive expression of BCL10 and the last one with continuous CD40 signaling (the L.CD40 model that we used as a control in **Figure 7**) (28, 49, 50). These three models are characterized by increased RelB activation. We previously demonstrated that immune surveillance may influence morphology and proliferation in the L.CD40 model. In this model, immunosuppression led to transformation of small indolent B-cell L.CD40 tumors into large B-cells with increased proliferation. Reactivating the anti-tumor response using anti-PD-L1 immunotherapy led to tumor regression (51, 52). These results on this mouse model suggest that the immunologically silent “indolent phenotype” of a B-cell tumor could be related to the immune pressure exerted on tumor B-cells. Whether and how activation of the alternative and canonical NF-κB pathways differently disturb immune surveillance remains to be determined, and comparison of both L.CD40 and Myd88^L252P^ mouse models could provide answers. However, as in the L.CD40 tumor model and in spleen marginal zone lymphomas (52, 53), the PD1/PD-L1 axis is most likely playing a role in the immune escape of aggressive tumor B-cells with MYD88 activation. Using Eµ-*MYC* transgenic hematopoietic stem cells (HSC) stably transduced with naturally occurring NF-κB mutants to generate various primary mouse lymphomas, Reimann et al. recently showed that MYD88 tumors express high levels of PD-L1 and that anti-PD-1 therapies induce T-cell dependent senescence of tumor cells (54). PD-L1 surface expression is weak or absent on WM tumor B-cells. However soluble PD-L1 serum levels are increased in WM patients and PD-L1 is upregulated by IL6 (55).

In summary, our longitudinal study of Myd88^L252P^ mice demonstrated that continuous MYD88 activation is able to promote early expansion of IgM LP cells and PCs with, first, serum polyclonal hyper Ig and then a monoclonal Ig peak. Ig peaks were constantly associated with B-cell lymphomas sharing characteristics with WM. Two major differences with WM were the spleen localization of Myd88^L252P^ tumors and increased proliferation. Here, we showed for the first time that IgM lymphoplasmacytic B-cell differentiation is at the heart of Myd88^L252P^ transforming potential. Thus, we also provide an interesting preclinical model for development of new therapeutic approaches or to study immune surveillance for example not only in WM but also in others B-cell lymphomas with features of plasma cell differentiation. Indeed, a better understanding of the underlying molecular mechanisms is necessary in order to develop new therapies for these incurable B-cell cancers.

## Data Availability Statement

The datasets presented in this study can be found in online repositories. The names of the repository/repositories and accession number(s) can be found below: https://www.ncbi.nlm.nih.gov/geo/, GSE138273.

## Ethics Statement

The studies involving human participants were reviewed and approved by the local IRB of the CHRU of Lille (CSTMT043). The patients/participants provided their written informed consent to participate in this study. The animal study was reviewed and approved by French national authorization number: 8708503 and French ethics committee registration number APAFIS#14581-2018041009469362 v3.

## Author Contributions

COu and LR contributed equally to this work. COu and LR performed and analyzed experiments. AS helped to perform the repertoire analysis. COb performed and analyzed ELISA. MD and NG performed the transcriptomic experiments. JF, SP, AS and LR performed the bioinformatics analyses. QL and CC performed flow cytometry analysis of bone marrow B-cell subsets. NF participated in the design of the project. KB and ME participated in the development of this study. CV-F created the mouse model, contributed to the experiments and analyzed the results. JF and CV-F directed the study and wrote the manuscript. All authors contributed to the article and approved the submitted version.

## Funding

The group of JF is supported by grants from the Ligue Nationale Contre le Cancer (Equipe labellisée Ligue), the Comité Orientation Recherche Cancer (CORC), the France Lymphome Espoir association, the Nouvelle Aquitaine Region and the Haute-Vienne and Corrèze committees of the Ligue Nationale Contre le Cancer. CV-F was supported by the France Lymphome Espoir association of patients. SP is supported by the Septentrion committee of Ligue contre le Cancer. ME is supported by an ANR @RAction grant (ANR-14-ACHN-0008), an ANR JCJC grant (ANR-19-CE15-0019-01), an IDEX Université de Paris grant, a Fondation Arthritis grant and a Fondation ARC grant (P JA20181208173). KB is supported by an ANR PRC grant (ANR-17-CE14-0019), an INCa grant (PRT-K 2017) and the Association Saint Louis pour la Recherche sur les Leucémies. 

## Conflict of Interest

The authors declare that the research was conducted in the absence of any commercial or financial relationships that could be construed as a potential conflict of interest.
