# Supplementary material for: Continuous MYD88 Activation Is Associated With Expansion and Then Transformation of IgM Differentiating Plasma Cells
Source: Front Immunol. 2021 May 4;12:641692. doi: 10.3389/fimmu.2021.641692 (PMC8129569; doi:10.3389/fimmu.2021.641692)
Supplement: Supplementary file 1 [file DataSheet_1.docx]

**Supplementary information for:**

**Continuous activation of MYD88 is associated with expansion and then transformation of IgM differentiating plasma cells.**

Catherine Ouk^1†^, Lilian Roland^1†^, Nathalie Gachard^1ɸ^, Stéphanie Poulain^2ɸ^, Christelle Oblet^1^, David Rizzo^1^, Alexis Saintamand^1^, Quentin Lemasson^1^, Claire Carrion^1^, Morgane Thomas^1^, Karl Balabanian^3^, Marion Espéli^3^, Marie Parrens^4^, Isabelle Soubeyran^5^, Mélanie Boulin^1^, Nathalie Faumont^1^, Jean Feuillard^1*^, Christelle Vincent-Fabert^1*^.

^1^ UMR CNRS 7276/INSERM U1262 CRIBL. University of Limoges, and hematology Laboratory of Dupuytren Hospital University Center (CHU) of Limoges; France.

^2^ UMR CANTHER «  CANcer Heterogeneity, Plasticity and Resistance to THERapies » INSERM 1277-CNRS 9020 UMRS 12. University of Lille., Hematology laboratory, Biology and pathology center, CHU de Lille, 59000 Lille, France.

^3^ Institut de Recherche Saint-Louis, EMiLy, INSERM U1160, University of Paris, France.

^4^ Pathology Department, Hospital University Center of Bordeaux, Bordeaux, France.

^5^ Laboratory of Pathology, Institut Bergonié, Bordeaux, France.

***Correspondings Authors:** Christelle Vincent-Fabert ([christelle.vincent-fabert@unilim.fr](mailto:christelle.vincent-fabert@unilim.fr)) and Jean Feuillard ( jean.feuillard@unilim.fr).

Supplementary information provided

- Supplementary Materials and Methods
- Additional references
- Supplementary Table 1: MYD88 and CXCR4 mutation status of the 11 WM patients from the series 1 for bone marrow B-cell
- Supplementary Table 2: Main clinical characteristics of patients
- Supplementary Tables 3-10: Excel File for transcriptome analysis
- Supplementary Figure legends (Supplementary Figures 1 to 9)

**Supplementary Materials and Methods**

**Sequence of the transgene**

**EcoRI AscI ClaI** Kozak

**GAATTCGGCGCGCCATCGAT**GCCGCCATGTCTGCGGGAGACCCCCGCGTGGGATCCGGGTCCCTGGACTCCTTCATGTTCTCCATACCCTTGGTCGCGCTTAACGTGGGAGTGAGGCGCCGCCTATCGCTGTTCTTGAACCCTCGGACGCCCGTGGCGGCCGACTGGACCTTGCTGGCGGAGGAGATGGGCTTCGAGTACTTGGAGATCCGAGAGCTGGAAACGCGCCCTGACCCCACTCGCAGTTTGTTGGATGCCTGGCAGGGGCGCTCTGGCGCGTCTGTCGGCAGGCTGCTAGAGCTGCTGGCCTTGTTAGACCGTGAGGATATACTGAAGGAGCTGAAGTCGCGCATCG**AGGAGGACTGCCAGAAATACTTAGGTAAGCAGCAGAACCAGGAGTCCGAGAAGCCTTTACAGGTGGCCAGAGTGGAAAGCAGTGTCCCACAAACAAAGGAACTGGGAGGCATCACCACCCTTGATGACCCCCTAG**GACAAACGCCGGAACTTTTCGATGCCTTTATCTGCTACTGCCCCAACGATATCGAGTTTGTGCAGGAGATGATCCGGCAACTAGAACAGACAGACTATCGGCTTAAGTTGTGTGTGTCCGACCGTGACGTCCTGCCGGGCACCTGTGTCTGGTCCATTGCCAGCGAGCTAATTGAGAAAAG**GTGTCGCCGCATGGTGGTGGTTGTTTCTGACGATTATCTACAGAGCAAGGAATGTGACTTCCAGACCAAGTTTGCACTCAGCCTGTCTCCAG**GTGTCCAACAGAAGCGACCGATTCCTATTAAATACAAGGCGATGAAGAAGGACTTTCCCAGTATCCTGCGGTTCATCACTATATGCGACTATACCAACCCTTGCACCAAGTCCTGGTTCTGGACCCGCCTTGCCAAGGCTTTGTCCCTGCCCTGA**TCGATGAAGTTCCTATTCCGAAGTTCCTATTCTCTAGAAAGTATAGGAACTTC**ctcgagggtaccccaattccgcccccCCCCCCCCCCTAACGTTACTGGCCGAAGCCGCTTGGAATAAGGCCGGTGTGCGTTTGTCTATATGTTATTTTCCACCATATTGCCGTCTTTTGGCAATGTGAGGGCCCGGAAACCTGGCCCTGTCTTCTTGACGAGCATTCCTAGGGGTCTTTCCCCTCTCGCCAAAGGAATGCAAGGTCTGTTGAATGTCGTGAAGGAAGCAGTTCCTCTGGAAGCTTCTTGAAGACAAACAACGTCTGTAGCGACCCTTTGCAGGCAGCGGAACCCCCCACCTGGCGACAGGTGCCTCTGCGGCCAAAAGCCACGTGTATAAGATACACCTGCAAAGGCGGCACAACCCCAGTGCCACGTTGTGAGTTGGATAGTTGTGGAAAGAGTCAAATGGCTCTCCTCAAGCGTATTCAACAAGGGGCTGAAGGATGCCCAGAAGGTACCCCATTGTATGGGATCTGATCTGGGGCCTCGGTGCACATGCTTTACATGTGTTTAGTCGAGGTTAAAAAACGTCTAGGCCCCCCGAACCACGGGGACGTGGTTTTCCTTTGAAAAACACGATGATAATATGGCCACAaccatgATGGTGAGCAAGGGCGAGGAGCTGTTCACCGGGGTGGTGCCCATCCTGGTCGAGCTGGACGGCGACGTAAACGGCCACAAGTTCAGCGTGTCCGGCGAGGGCGAGGGCGATGCCACCTACGGCAAGCTGACCCTGAAGTTCATCTGCACCACCGGCAAGCTGCCCGTGCCCTGGCCCACCCTCGTGACCACCTTCGGCTACGGCCTGATGTGCTTCGCCCGCTACCCCGACCACATGAAGCAGCACGACTTCTTCAAGTCCGCCATGCCCGAAGGCTACGTCCAGGAGCGCACCATCTTCTTCAAGGACGACGGCAACTACAAGACCCGCGCCGAGGTGAAGTTCGAGGGCGACACCCTGGTGAACCGCATCGAGCTGAAGGGCATCGACTTCAAGGAGGACGGCAACATCCTGGGGCACAAGCTGGAGTACAACTACAACAGCCACAACGTCTATATCATGGCCGACAAGCAGAAGAACGGCATCAAGGTGAACTTCAAGATCCGCCACAACATCGAGGACGGCAGCGTGCAGCTCGCCGACCACTACCAGCAGAACACCCCCATCGGCGACGGCCCCGTGCTGCTGCCCGACAACCACTACCTGAGCTACCAGTCCGCCCTGAGCAAAGACCCCAACGAGAAGCGCGATCACATGGTCCTGCTGGAGTTCGTGACCGCCGCCGGGATCACTCTCGGCATGGACGAGCTGTACAAGTAAtaatccgggatccggagagctcccaac**GAAGTTCCTATTCCGAAGTTCCTATTCTCTAGAAAGTATAGGAACTTC**CTCGAGGTTGGATGCAGCCCGGGGGATCCACTAGTTCTAGAGCTGTGCCTTCTAGTTGCCAGCCATCTGTTGTTTGCCCCTCCCCCGTGCCTTCCTTGACCCTGGAAGGTGCCACTCCCACTGTCCTTTCCTAATAAAATGAGGAAATTGCATCGCATTGTCTGAGTAGGTGTCATTCTATTCTGGGGGGTGGGGTGGGGCAGGACAGCAAGGGGGAGGATTGGGAAGACAATAGCAGGCATGCTGGGGATGCGGTGGGCTCTATGG**GGCGCGCCGCGGCCGC**

MYD88

IRES

**FRT**

BGH poly A

YFP

**ClaI**

mutation

**FRT**

**AscI NotI**

**Screening of ES clones and mice**

Primers sequences for screening ES cells and transgenic mice were: 1) for recombined allele, 5pROSA_arm: 5’-CGCCTAAAGAAGAGGCTGTG-3’ ; neo1: 5’-GGA TGA TCT GGA CGA AGA GC-3’, 2) for WT allele, Rosa_fw: 5’-CTC TCC CAA AGT CGC TCT G-3’ ; Rosa_rev: 5’-TAC TCC GAG GCG GAT CAC AAG C-3’ ; 3) for CD19_Cre allele, CD19c: 5’-AAC CAG TCA ACA CCC TTC C-3’ ; CD19d: 5’-CCA GAC TAG ATA CAG ACC AG-3’ ; CD19Cre7: 5’-TCA GCT ACA CCA GAG ACG G-3’.

**Flow cytometry**

For bone marrow analysis, antibodies used were: CD19 BV510 (clone 1D3, BD Biosciences, San Jose, California), CD21 PECF594 (clone 7G6, BD Biosciences, San Jose, California), CD23 BV711 (clone B3B4, BD Biosciences, San Jose, California), IgM PC7 (clone eB121-15F9, Invitrogen), CD38 APC-R700 (clone 90, Invitrogen), CD138 APC-R700 (clone 281-2, Biolegend, San Diego, California), CD93 BV650 (clone AA4.1, BD Biosciences, San Jose, California), B220 APC (CloneRA3-6B2, Biolegend, San Diego, California).

For splenocytes, antibodies used were: CD80 APC, (clone 16-10A1, Biolegend , San Diego, California), CD86 PE-Cy7 (clone GL-1, Biolegend , San Diego, California), CD3 APC (clone 17 A2, Biolegend , San Diego, California), CD45R/B220 BV421 (clone RA3-6B2, Biolegend, San Diego, California), CD138 APC-R700 (clone 281-2, BD Biosciences, San Jose, California).

**Bioinformatics analysis of transcriptomes**

All analyses were performed with the version 1.1.463 and 3.6.1 of RStudio and R (Free Software Foundation, Inc, Boston, MA). RMA normalization was done with the “oligo” R package (1). This resulted in a table giving the normalized signal intensity of 54675 and 770069 probesets for Human Genome U133 Plus 2.0 and MoGene-2_1-st-v1 chips respectively, the latter corresponding to 41345 transcripts. After filtering invariant and unannotated probesets, differential analysis was performed with the “Limma” R package (2). For the MoGene-2_1-st-v1 chip, signal levels of selected probesets were mean-aggregated according the Affymetrix transcript_cluster_id. Unsupervised clustering were performed with the LPS package. Mouse plasma cell signatures were issued from (3) as well as from GSEA website (<http://software.broadinstitute.org/gsea/index.jsp>) (4,5). Unsupervised clustering and Linear Predicting Score were done using the LPS package (<https://bioinformatics.ovsa.fr/LPS> and (6)).

**Repertoire analysis**

Primers were: 1) for membrane µ exon, Mm_IgM_Memb_rev: 5’-TTC CTC CTC AGC ATT CAC CT-3’ ; 2) for membrane γ exon, Mm_IgG_Memb_rev : 5’-GCT GAT GAA GAT GGT GAT GG-3’ ; 3) for secreted µ exon, Mm_IgM_Sec_rev: 5’-CGC TAG CAT GGT CAA TAG CA-3’ ; 4) for secreted γ exons, Mm_IgG1_Sec_rev : 5’-CAA GGA CAC TGG GAT CAT TTA C-3’, Mm_IgG2A-B_Sec_rev : 5’-TGG GTG CTG AGC TCA TTT AC-3’, Mm_IgG2C_Sec_rev : 5’-GAA AGA ACC AGG ACA GTT TTA C-3’, Mm_IgG3_Sec_rev : 5’-CTA GGT GCT GTT CTC ATT TAC-3’ .

**Additional references**

1. Carvalho BS, Irizarry RA. A framework for oligonucleotide microarray preprocessing. Bioinformatics. 1 oct 2010;26(19):2363‑7.

2. Ritchie ME, Phipson B, Wu D, Hu Y, Law CW, Shi W, et al. limma powers differential expression analyses for RNA-sequencing and microarray studies. Nucleic Acids Res. 20 avr 2015;43(7):e47.

3. Shi W, Liao Y, Willis SN, Taubenheim N, Inouye M, Tarlinton DM, et al. Transcriptional profiling of mouse B cell terminal differentiation defines a signature for antibody-secreting plasma cells. Nat Immunol. juin 2015;16(6):663‑73.

4. Mootha VK, Lindgren CM, Eriksson K-F, Subramanian A, Sihag S, Lehar J, et al. PGC-1alpha-responsive genes involved in oxidative phosphorylation are coordinately downregulated in human diabetes. Nat Genet. juill 2003;34(3):267‑73.

5. Subramanian A, Tamayo P, Mootha VK, Mukherjee S, Ebert BL, Gillette MA, et al. Gene set enrichment analysis: a knowledge-based approach for interpreting genome-wide expression profiles. Proc Natl Acad Sci USA. 25 oct 2005;102(43):15545‑50.

6. Mareschal S, Dubois S, Viailly P-J, Bertrand P, Bohers E, Maingonnat C, et al. Whole exome sequencing of relapsed/refractory patients expands the repertoire of somatic mutations in diffuse large B-cell lymphoma. Genes Chromosomes Cancer. mars 2016;55(3):251‑67.

**Supplementary Table 1: MYD88 and CXCR4 mutation status of the 11 WM patients for bone marrow B-cell**

| **UPN** | **Sex** | **MYD88 L265P** | **CXCR4 mutation** |
| --- | --- | --- | --- |
| # 1 | Male | Positive | Negative |
| # 2 | Female | Positive | Negative |
| # 4 | Male | Positive | Negative |
| # 5 | Female | Positive | Positive |
| # 9 | Male | Positive | Negative |
| # 12 | Male | Positive | Negative |
| # 13 | Male | Positive | Positive |
| # 20 | Male | Positive | Negative |
| # 26 | Male | Positive | Negative |
| # 27 | Female | Positive | Negative |
| # 31 | Female | Positive | Positive |

UPN : unique patient number

**Supplementary Table 2: main clinical characteristics of patients**

| **Dg** | **N** | **Age**  **(mean +/- SD)** | **Sex ratio** | **Immunoglobulin Peak** | **Bone Marrow involvement** | **Spleen enlargment** | **CXCR4 mutation** |
| --- | --- | --- | --- | --- | --- | --- | --- |
| WM *MYD88^mut^* | 15 | 65.2+/- 9.7 | 4 | 100% | 100% | 27% (4/15) | 18% |
| WM/LPL *MYD88^wt^* | 5 | 65.5 +/- 4.5 | 5 | 80% | 60% (3/5) | 20% (1/5) |  |
| CLL | 19 | 65.9 +/- 12.3 | 2.2 | 11% | NE | 67% (6/19) |  |
| NMZL | 12 | 67.6 +/- 11 | 1.4 | 50% | 37% (4/12) | 37% (4/12) |  |
| FCL | 4 | 60.3 +/- 3 | 3 |  |  |  |  |
| FH | 3 | 38.3 +/- 14.6 | 0.33 |  |  |  |  |

Dg : diagnosis ; N : number of cases ; SD : standard deviation ; WM : Waldentröm Macroglobulinemia ; LPL : lymphoplasmacytic lymphoma ; CLL : chronic lymphocytic leukemia ; NMZL : nodal marginal zone lymphoma ; FCL : follicular cell lymphoma ; FH : benign follicular hyperplasia ; *MYD88*^mut^: mutated *MYD88* gene ; *MYD88^w^* : wild type *MYD88* gene ; NE : not evaluated.

**Supplementary Tables 3-10: Excel File for transcriptome analysis**

**Supplementary Figure legends (Supplementary Figures 1 to 9)**

**Supplementary Figure 1: *In vitro* model with expression of *Myd88^L252P^*-IRES-*Yfp* insert.** (A) Schematic representation of the *Myd88^L252P^*-IRES-*Yfp* insert: the *Myd88^L252P^* sequence was in frame with the Internal Ribosomal Entry Site (IRES) and the coding sequence for Yellow Fluorescent Protein (*Yfp*). This 2.6 kB sequence was directly synthetized and cloned within the pcDNA3.1 vector upstream from the pCMV promoter (pcDNA3.1/*Myd88^L252P^* vector). As a control the same construct with the wild type Myd88 sequence was built (pcDNA3.1/*Myd88^WT^* vector). (B): MYD88 protein expression after transfection of A20 murine B-cells. Empty pcDNA3.1 (Ctrl), pcDNA3.1/*Myd88^L252P^* (L252P) and pcDNA3.1/*Myd88^WT^* (WT) vectors were transiently transfected into A20 murine B-cells. MYD88 protein expression of non-transfected (NT) and transfected cells was assessed by Western blot. Revelation of tubulin was used as a loading protein control. (C) Flow cytometry detection of YFP 48H after transfection of A20 murine B-cells with either the empty pcDNA3.1 (Ctrl), pcDNA3.1/*Myd88^L252P^* (L252P) and pcDNA3.1/*Myd88^WT^* (WT) vectors. Percentages of positive cells are indicated in each histogram. (D) Luciferase gene reporter assay: A20 murine B cells were co transfected with either the empty pcDNA3.1 (Ctrl) , pcDNA3.1/*Myd88^L252P^ (*L252P) and pcDNA3.1/*Myd88^WT^* (WT) vectors and the *Renilla luciferase* vector (pRL-TK) which harbors the luciferase reporter gene downstream from the CMH class I NF-kappa B binding site (3X-κB-L) or its mutated inactive variant (3X-mutκB-L). (E) Targeting strategy for insertion of a conditional *Myd88^L252P^*-IRES-*Y*fp (Myd88^L252P-flSTOP^) into the murine *Rosa26* locus. This strategy was similar to the one published by Hömig-Hölzel et al (41). Briefly, CRE mediated recombination leads to deletion of the stop cassette and expression of the *Myd88^L252P^*-IRES-*Y*fp insert (Myd88^L252P^ mice) under transcriptional control of the endogenous *Rosa26* promoter. STOP: Stop cassette. (F) Flow cytometry detection of YFP in T and B cells from peripheral blood (upper panel) or splenocytes (lower panel) of 6 months old Myd88^L252P^ mice. Gating done on CD3 positive and B220 positive lymphocytes for T and B cells respectively. (G) *Tnfaip3* expression analysis by quantitative RT-PCR in splenocytes. mRNA levels from 12 months-old Myd88^L252P^ mice compared to their LMC Cd19^Cre^ were normalised to *Hprt* expression (n=3) for each group. Results are expressed as the mean ± SEM. Mann Whitney test p-value <0.05 is symbolized by *.

**Supplementary Figure 2:** IgM and IgG serum levels in Cd19^Cre^ (n=15) and Myd88^L252P^ mice (n=36) over age. Results are expressed as the mean ± SEM. Mann Whitney test p-value <0.05, < 0.01 and < 0.001 are symbolized by *, ** and *** respectively.

**Supplementary Figure 3: example of the gating strategy used for the bone marrow analysis by flow cytometry related to Figure 3.**

Cell debris were first eliminated on a FSC/SSC 2-dimensional plot. Then, following the general analysis principles previously reported (Faucher et al., Cytometry A, 2007), the gating strategy was based on orientating and specific gates.

Gating was on CD19 positive, B220 positive or CD138 positive events. From these gates, two Boolean gates named “B220^pos^ OR CD19^pos^ OR CD138^pos^” and “CD19^pos^ OR CD138^pos^“ were set up. Total PCs were counted on a specific gate from CD138^high^ B220^neg^ events. IgM PCs were gated from total PCs.

A specific hinged quadrant was set up on the IgM/CD138 2-dimensional plot gated on “CD19^pos^ OR CD138^pos^”. This quadrant was also used to color IgM^low^/CD138^neg^, IgM^high^/CD138^neg^, IgM^high^/CD138^pos^, IgM^low^/CD138^pos^ events in blue, orange, red and purple respectively.

One example of such gating is given for each genotype Cd19^CRE^ (A) and Myd88^L252P^ (B).

**Supplementary Figure 4: Analysis of the B-cell differentiation according the expression of the MYD88 L252P mutation in Myd88L252P mice.** This analysis show that Myd88^L252P^ CD19^pos^/YFP^pos^ B cells (in yellow) tended to be accumulated into the IgM^high^ B-cell compartment. The CD19^pos^/YFP^neg^ population is represented in blue.

**Supplementary Figure 5: Distribution of spleen B/T cell ratio from aged Cd19^Cre^ (n=9) and Myd88^L252P^(n=14) mice (≥ 32 weeks old).** Results are expressed as the mean ± SEM. Mann Whitney test p-value <0.001 is symbolized by ***.

**Supplementary Figure 6: Cytological aspect of spleen imprints from one Cd19^Cre^ (left) and two Myd88^L252P-YFP^ mice.** Cells from Myd88^L252P^ spleen exhibited marked lymphoplasmacytic morphology. Examples of these lymphoplasmacytic cells are surrounded by dashed black lines.

**Supplementary Figure 7: example of the gating strategy used for the spleen analysis by flow cytometry.** (A) Example of the strategy from a Cd19^CRE^ mouse. Cell debris were first eliminated on a FSC/SSC 2-dimensional plot and doublets were excluded with a SSC-A/SSC-H 2-dimensional plot. Then, with a CD19/CD138 2-dimensional plot a first specific gate defined the B cell and plasmocytes population (BLs and PCs). From this gate, specific gates for MZ B cells, follicular cells, other B cells and PCs were drawn. Finally, CD93^neg^/IgM^pos^ PCs were gated from total PCs. (B) Example of the strategy from a Myd88^L252P^ mouse. The same strategy was adopted as the used for the Cd19^CRE^ mice except for an additional gate of YFP positive cells defined among the BL and PC population.

**Supplementary Figure 8: *In vivo* proliferation index of spleen B cells from Cd19^Cre^ and Myd88^L252P^ mice.** BrdU was intra peritoneally injected 18 hours before sacrifice. Percentage of BrdU positive B cells was assessed by flow cytometry. The red square represents a mouse with a tumor in transformation whereas black squares indicate mice with LPL phenotype. Wilcoxon’s test p-value < 0.05 is symbolized by *.

**Supplemental Figure 9: Aggregation of the 40 Kmean clusters generated from the 1515 differentially expressed genes betweed Cd19^Cre^ and Myd88^L252P^ mice:** total mRNA was extracted from whole spleen tissues. Gene expression profiles were obtained using the MoGene-2_1-st-v1 Affymetrix chip. Three thousand two hundred thirty six mRNA transcripts were selected to be differentially expressed using the Limma R package. Genes that were too heterogeneously expressed were eliminated, resulting in a final selection of 1515 genes. These genes were partitioned in 40 Kmean clusters. Each Kmean cluster was taken as a metagene, corresponding to the expression mean of the genes from a given cluster for each sample. An Euclidean distance between each metagene was calculated after principal component analysis using the first two components. The two closest Kmean clusters (metagene or mean vectors) were merged. This was repeated until maximization of the absolute value of Chi2 (Faumont et al, J Virol 2009). This resulted in 14 aggregated clusters. Functional annotation of the aggregated Kmean clusters was performed using the Ingenuity Pathway Analysis (IPA) Software.

Left panel: principal component analysis (upper) and heatmap with its hierarchical clustering (lower) of the 40 Kmean clusters taken as metagenes. On the periphery of the PCA graph and on the right of the heatmap are the arbitrary numbers of the 40 Kmean clusters.

Right panel: principal component analysis (upper) and heatmap with its hierarchical clustering (lower) of the 14 aggregated Kmean clusters taken as metagenes. On the periphery of the PCA graph and on the right of the heatmap are the list of Kmean clusters aggregated together.
