# Supplementary material for: Continuous MYD88 Activation Is Associated With Expansion and Then Transformation of IgM Differentiating Plasma Cells
Source: Front Immunol. 2021 May 4;12:641692. doi: 10.3389/fimmu.2021.641692 (PMC8129569; doi:10.3389/fimmu.2021.641692)
Supplement: Supplementary file 3 [file Presentation_1.pptx]

## Slide 1
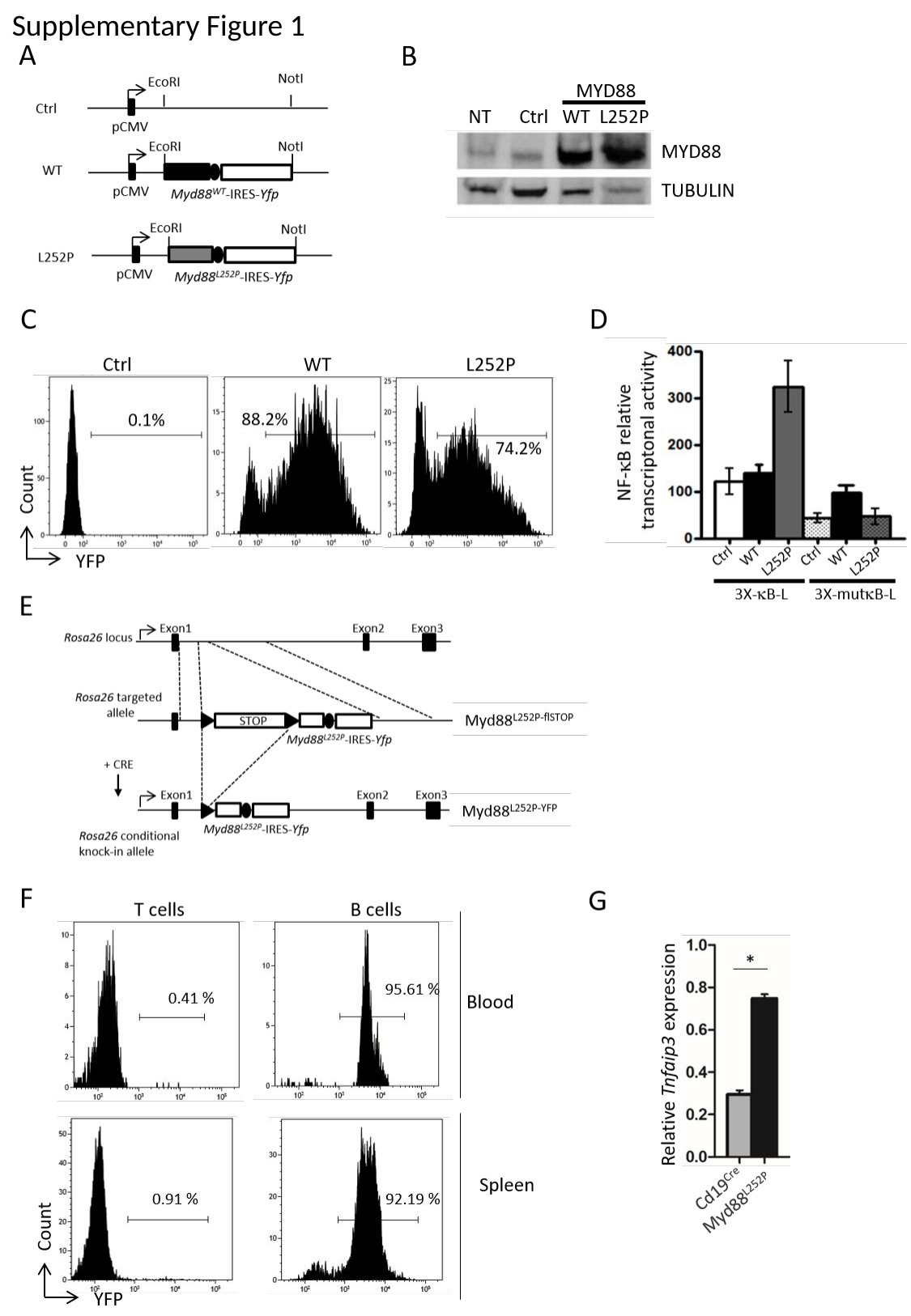

Supplementary Figure 1

## Slide 2
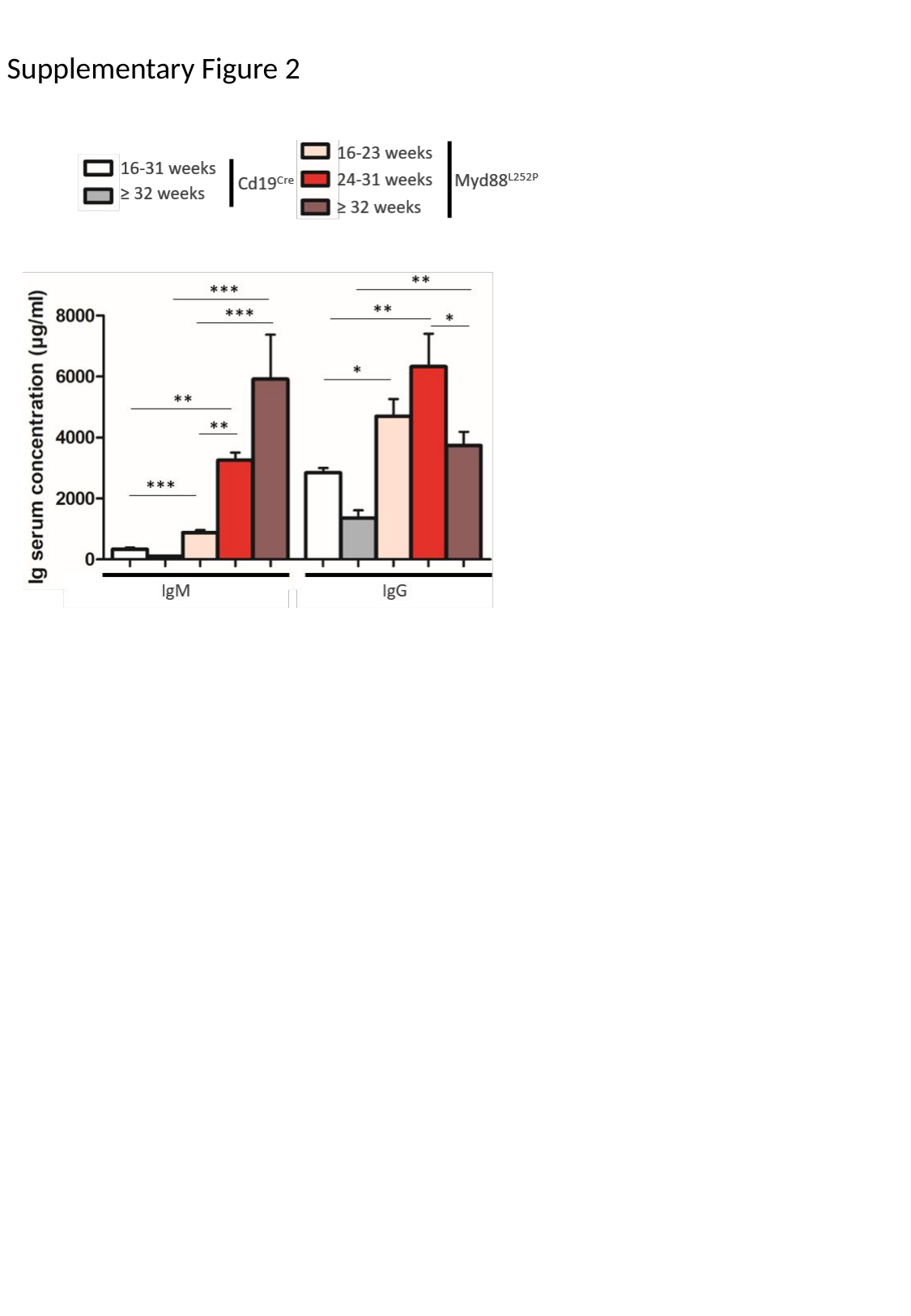

Supplementary Figure 2

## Slide 3
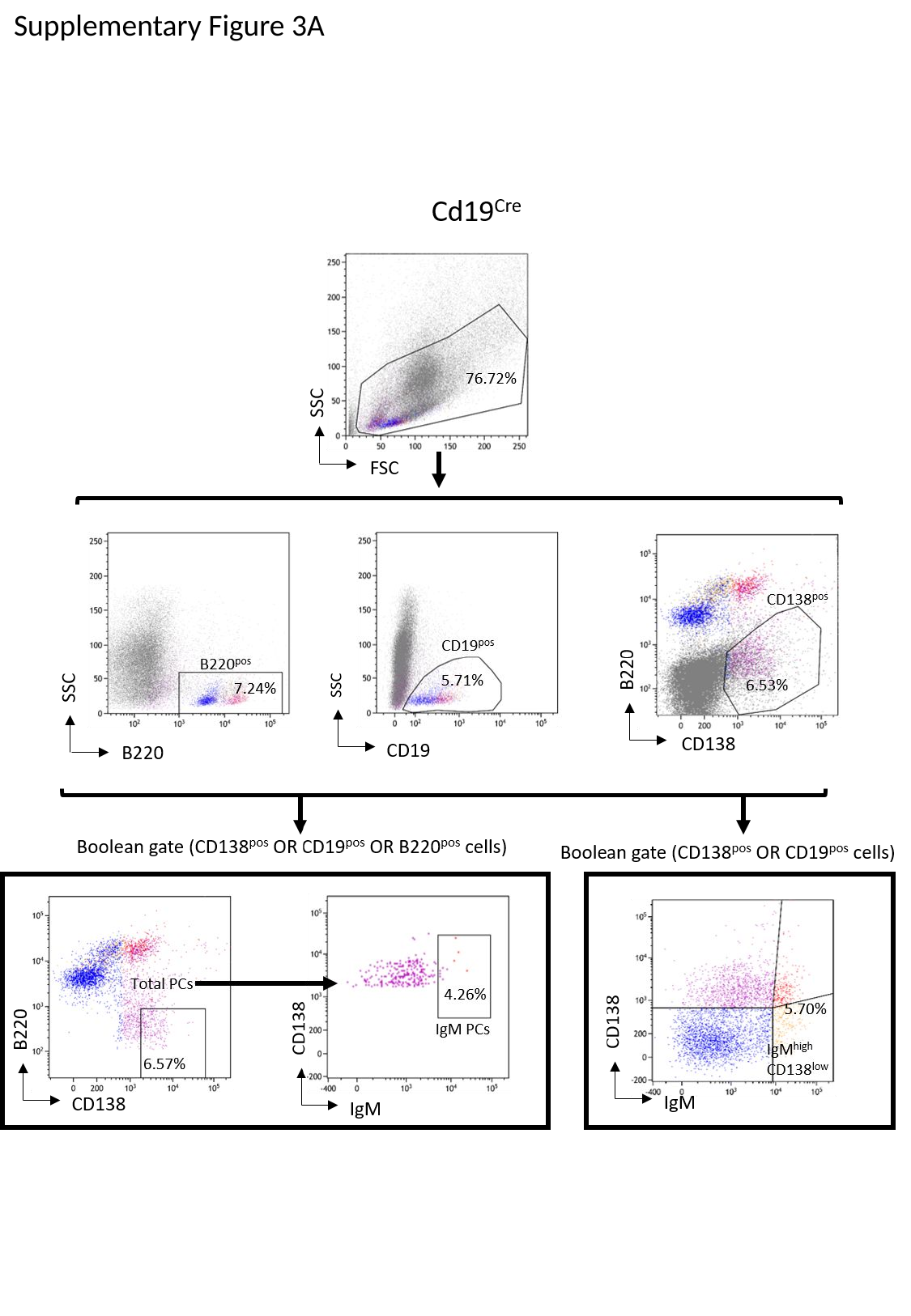

Supplementary Figure 3A

## Slide 4
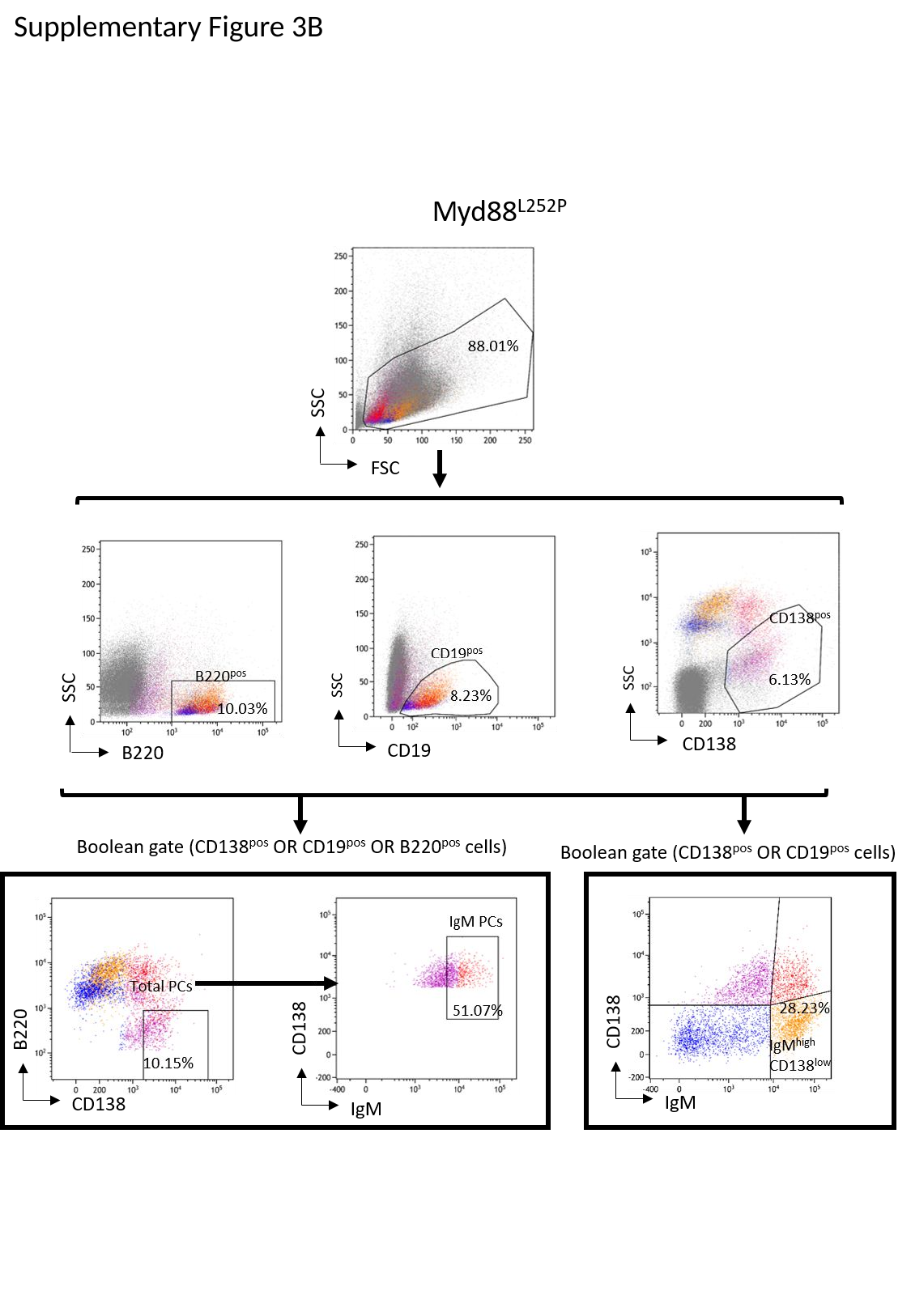

Supplementary Figure 3B

## Slide 5
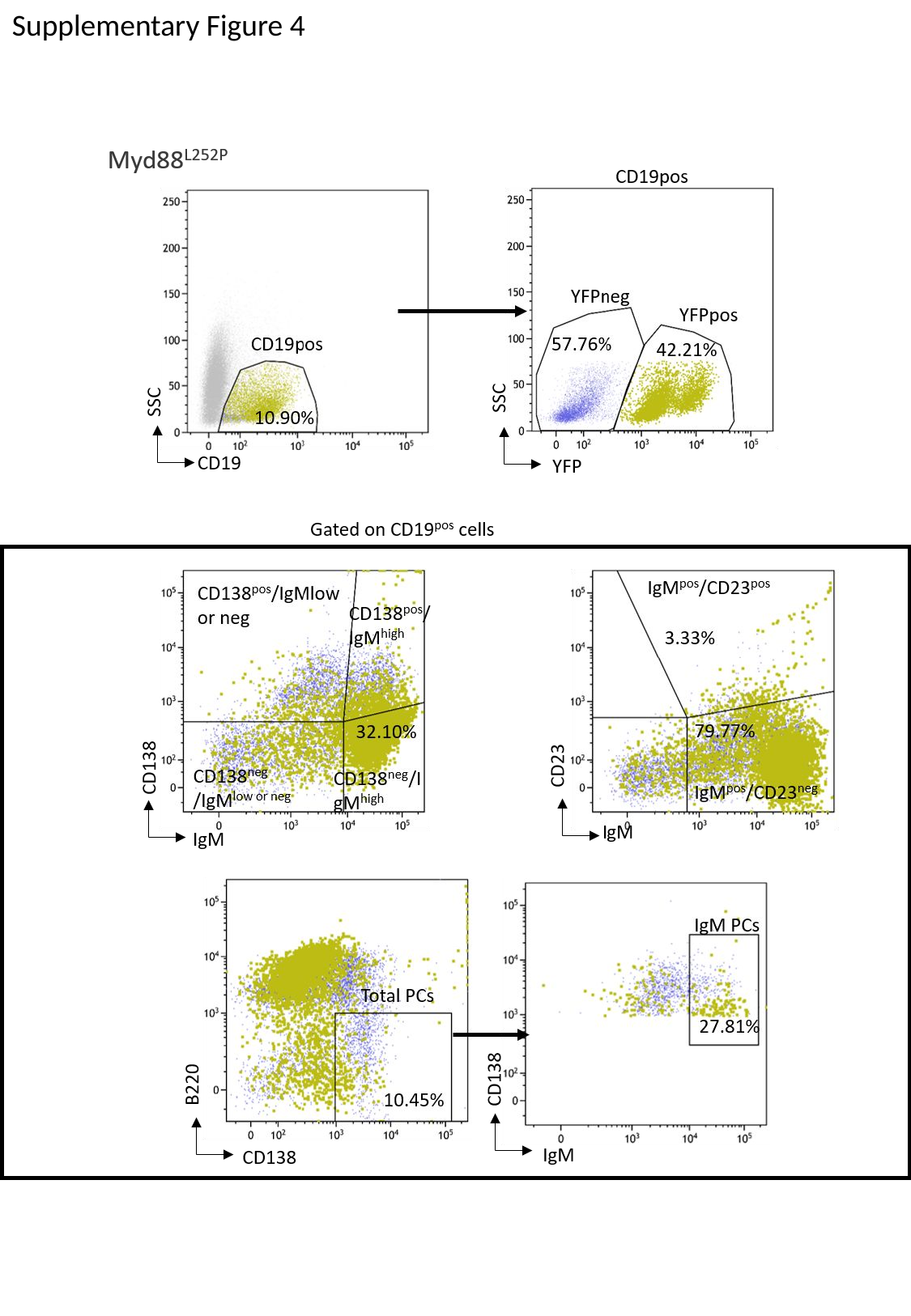

Supplementary Figure 4

## Slide 6
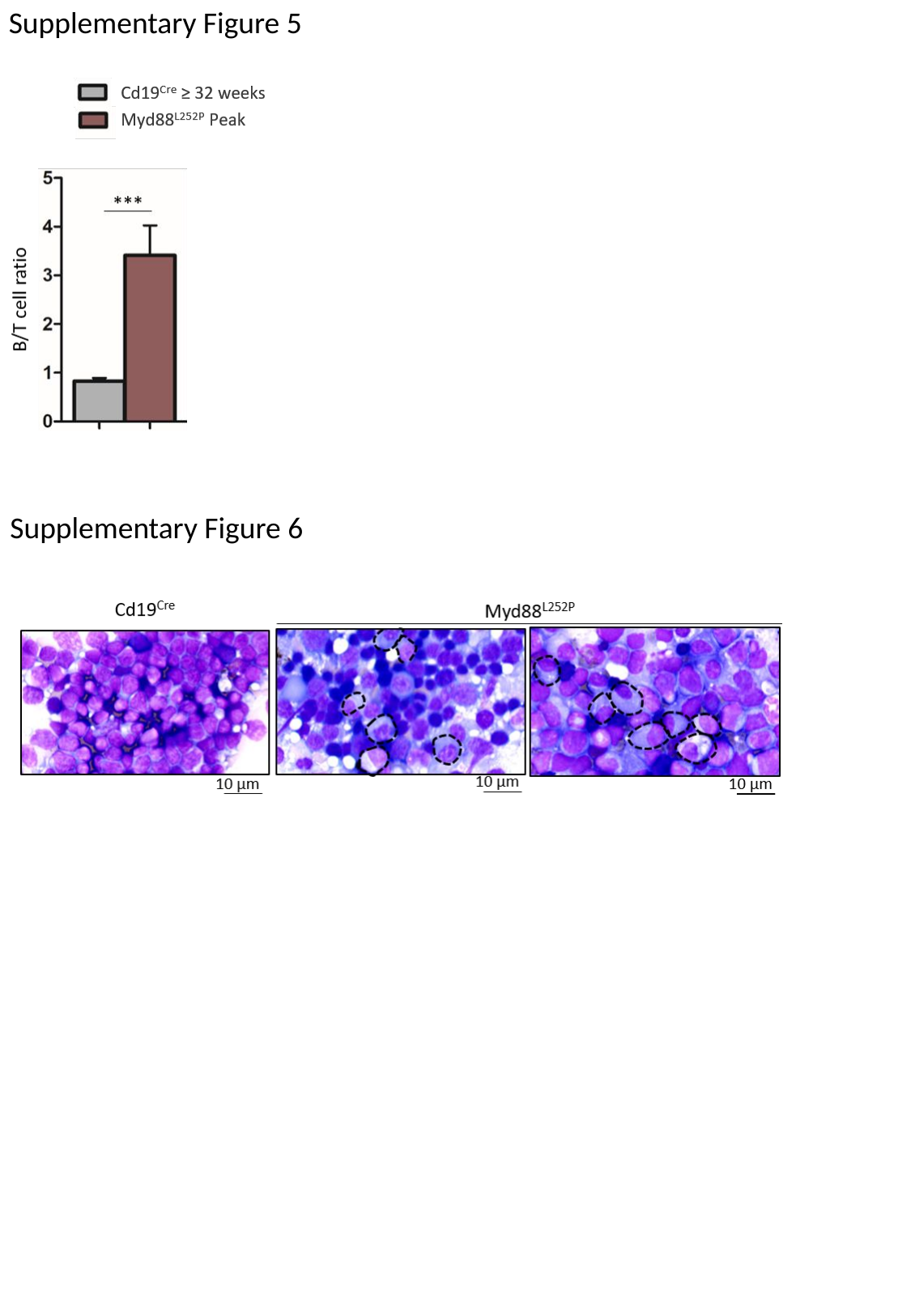

Supplementary Figure 5
Supplementary Figure 6

## Slide 7
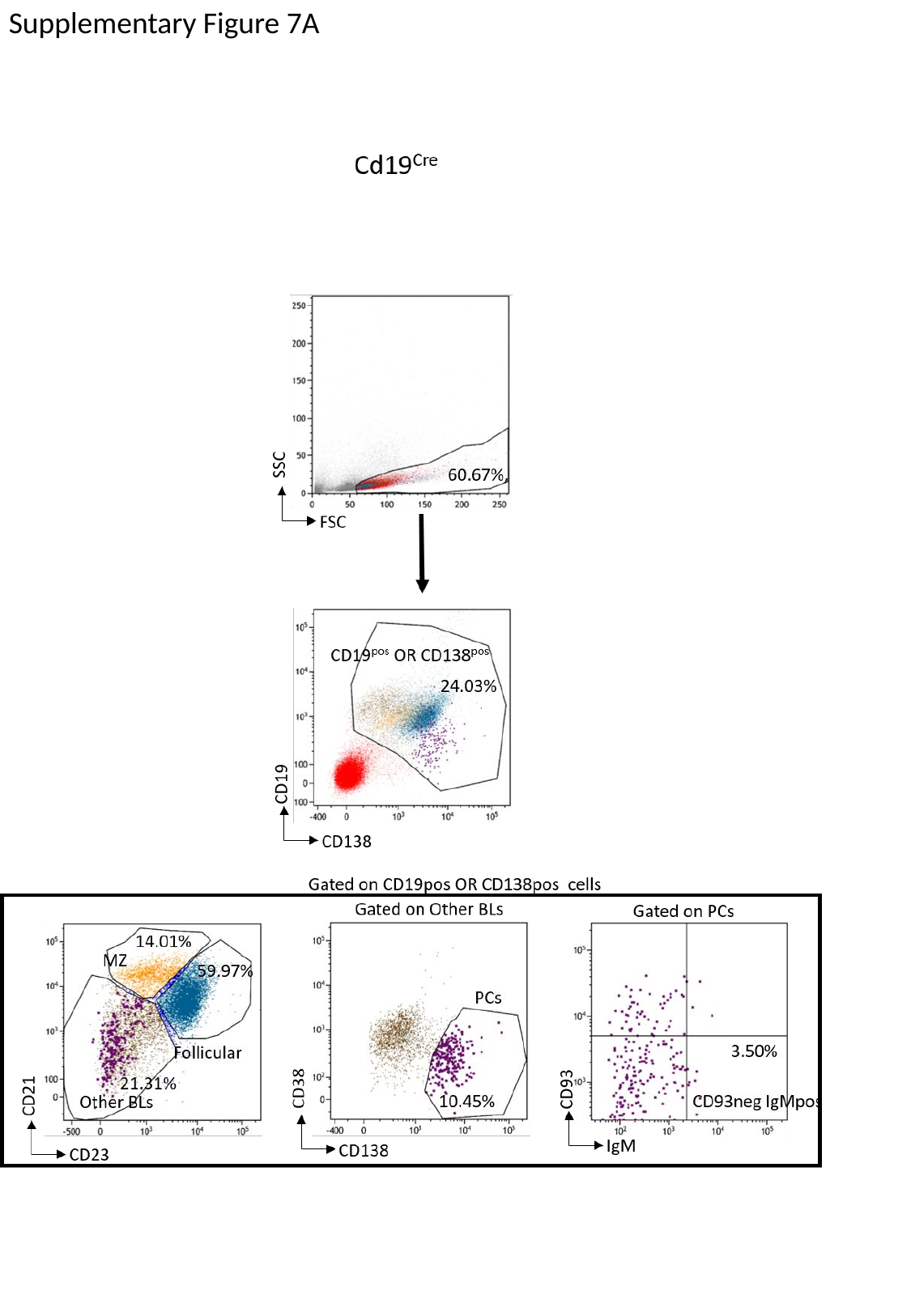

Supplementary Figure 7A

## Slide 8
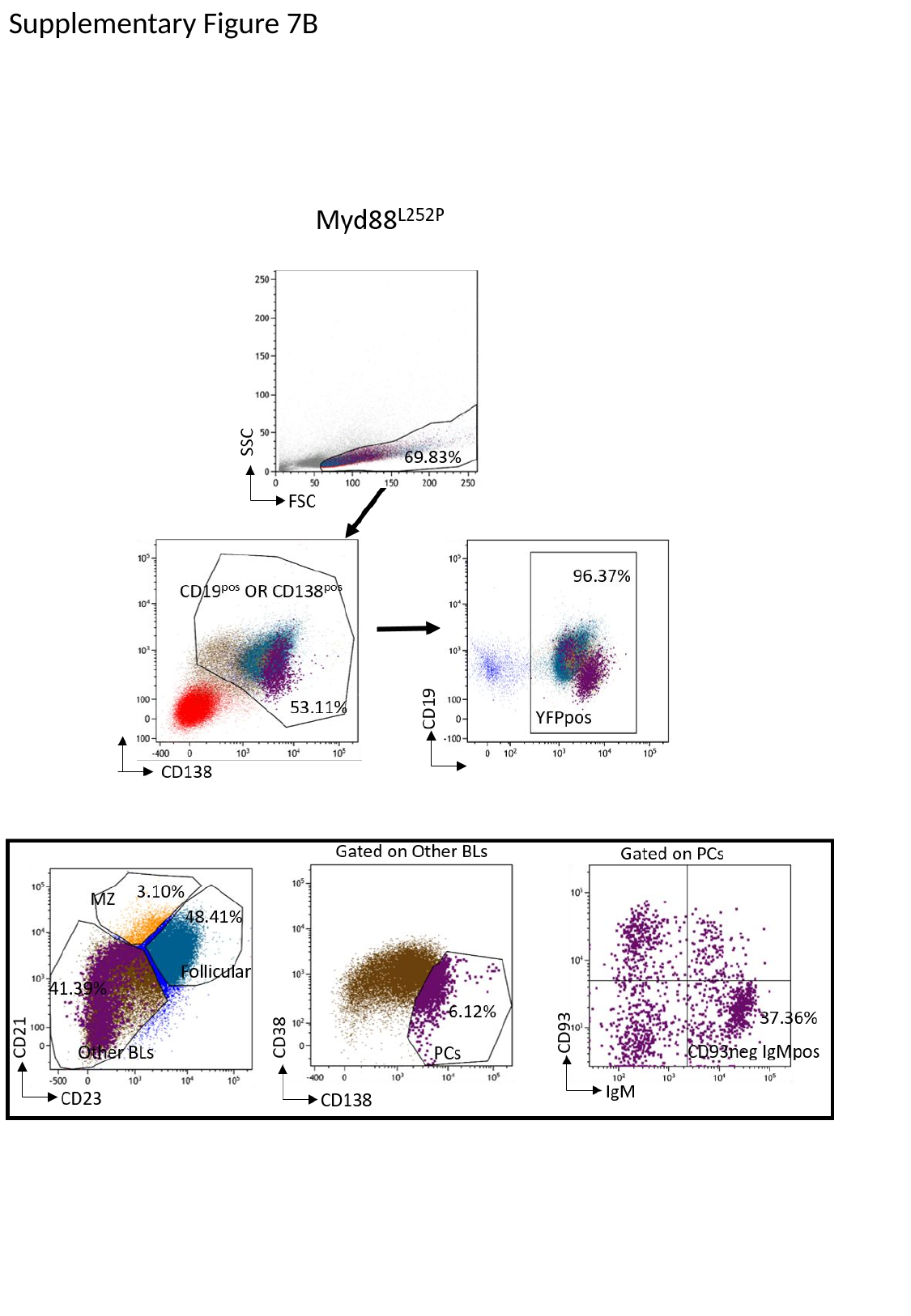

Supplementary Figure 7B

## Slide 9
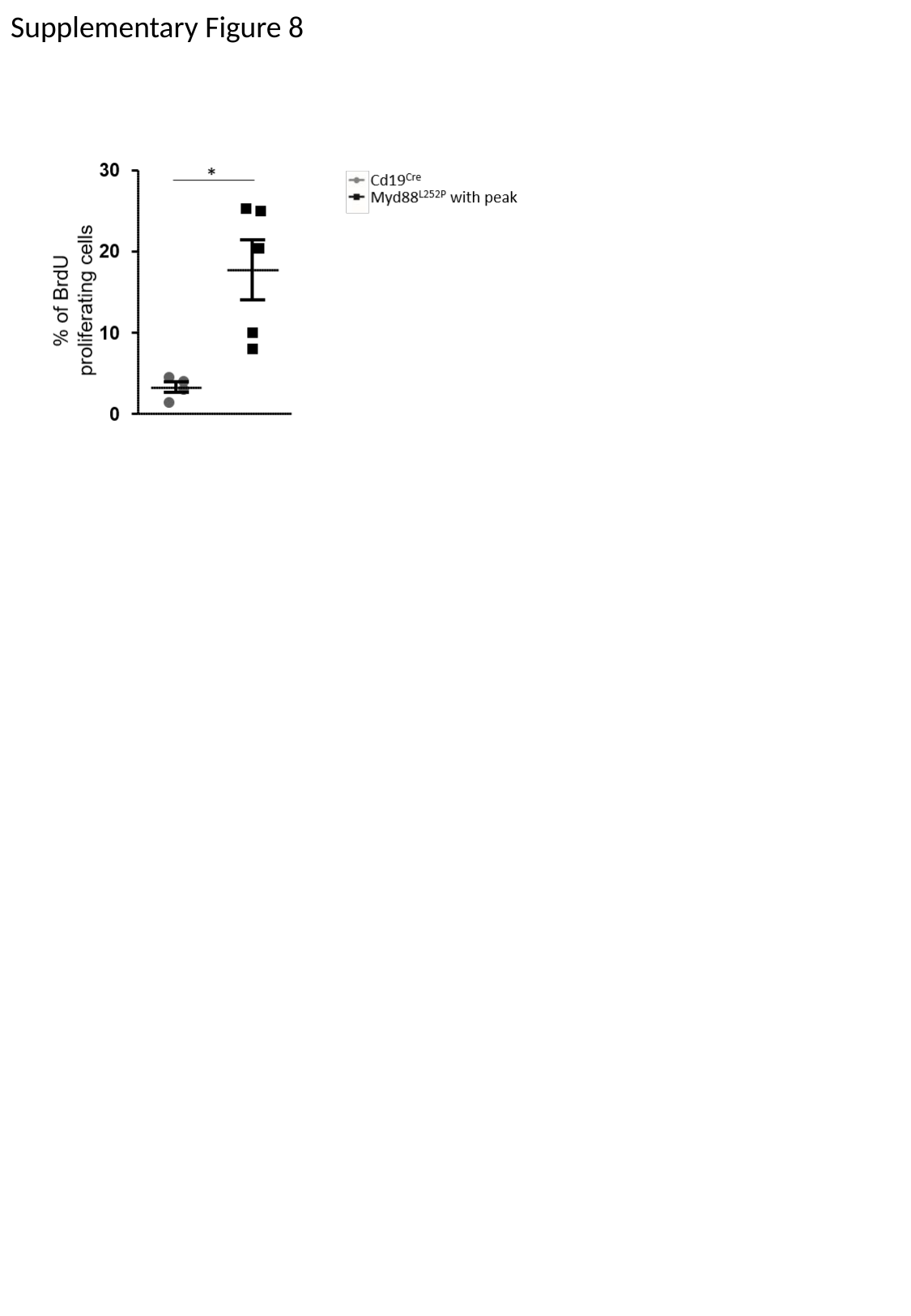

Supplementary Figure 8

## Slide 10
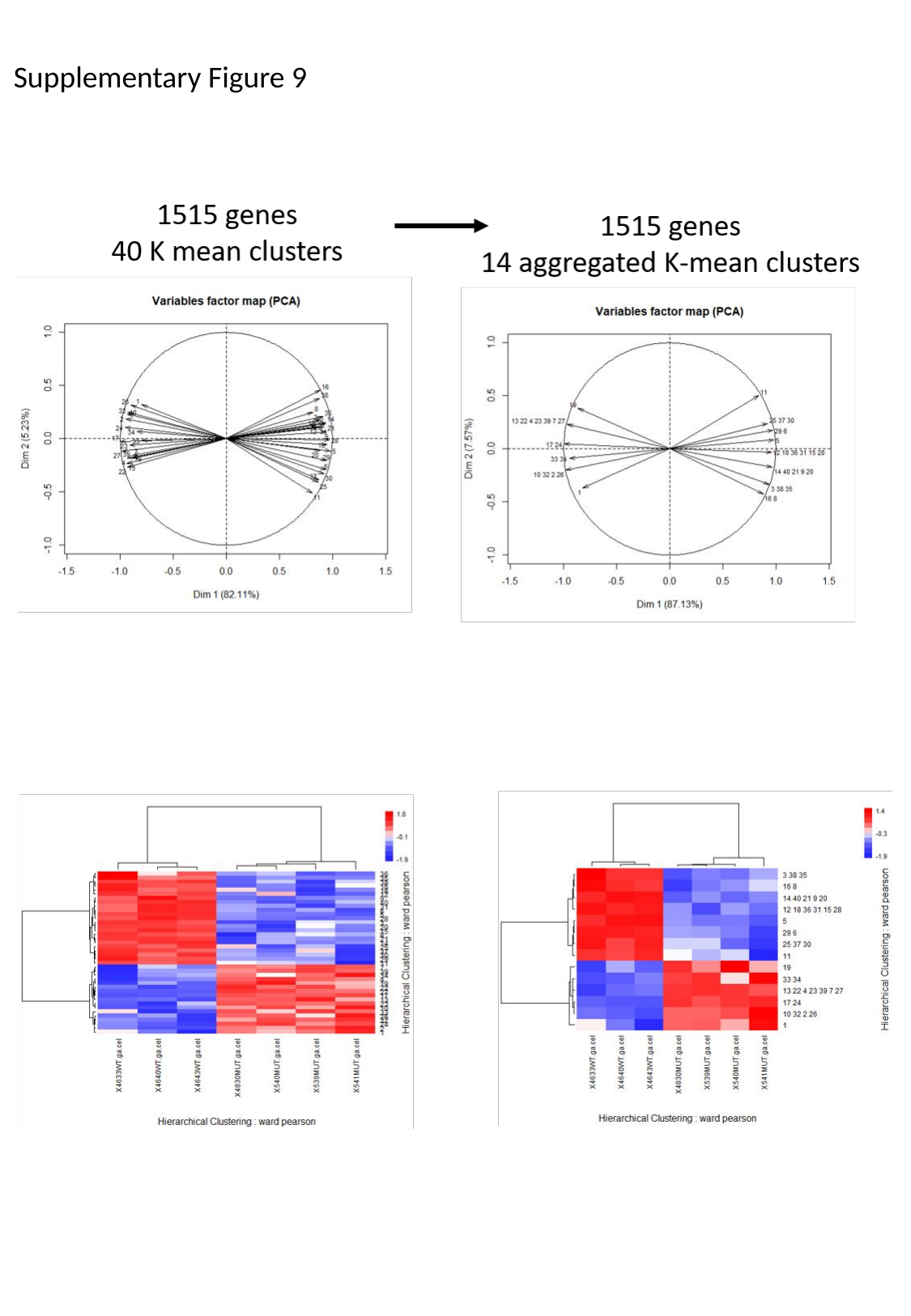

Supplementary Figure 9
